# m6A-Modified Nucleotide Bases Improve Translation of In Vitro-Transcribed Chimeric Antigen Receptor (CAR) mRNA in T Cells

**DOI:** 10.3390/ijms27020796

**Published:** 2026-01-13

**Authors:** Nga Lao, Simeng Li, Marina Ainciburu, Niall Barron

**Affiliations:** 1 National Institute for Bioprocessing Research and Training, Blackrock, A94 X099 Dublin, Ireland; 2 School of Chemical and Bioprocess Engineering, University College Dublin, Belfield, D04 V1W8 Dublin, Ireland

**Keywords:** transient CAR-T, RNA therapy, cell therapy, m6A, in vitro transcription, T cell engineering, UTR engineering

## Abstract

Lentiviral transduction remains the gold standard in adoptive modified cellular therapy, such as CAR-T; however, genome integration is not always desirable, such as when treating non-fatal autoimmune disease or for additional editing steps using CRISPR to produce allogeneic CAR-modified cells. Delivering in vitro-transcribed (IVT) mRNA represents an alternative solution but the labile nature of mRNA has led to efforts to improve half-life and translation efficiencies using a range of approaches including chemical and structural modifications. In this study, we explore the role of N^6^–methyladenosine (m6A) in a CD19-CAR sequence when delivered to T cells as an IVT mRNA. In silico analysis predicted the presence of four m6A consensus (DRACH) motifs in the CAR coding sequence and treating T cells with an inhibitor of the m6A methyltransferase (METTL3) resulted in a significant reduction in CAR protein expression. RNA analysis confirmed m6A bases at three of the predicted sites, indicating that the modification occurs independently of nuclear transcription. Synonymous mutation of the DRACH sites reduced the levels of CAR protein from 15 to >50% depending on the T cell donor. We also tested a panel of CAR transcripts with different UTRs, some containing m6A consensus motifs, and identified those which further improved protein expression. Furthermore, we found that the methylation of consensus m6A sites seems to be somewhat sequence-context-dependent. These findings demonstrate the importance of the m6A modification in stabilising and enhancing expression from IVT-derived mRNA and that this occurs within the cell, meaning targeted in vitro chemical modification during mRNA manufacturing may not be necessary.

## 1. Introduction

The success of the novel vaccines during the Covid pandemic has accelerated interest in utilising mRNA in applications other than just vaccines, for example, in protein replacement therapies [1] or modified cell therapies [2]. Modified cell therapy involves reprogramming immune cells (mainly T cells) to target specific tumour antigens and has revolutionised cancer treatment since Emily Whitehead, the first child to successfully receive CAR-T treatment, was cured of acute leukaemia in 2012 [3]. At present, the six CAR-T products that have been approved by the FDA for the treatment of several forms of blood cancer [4] use either Lentivirus or retrovirus to deliver the CAR. Although the parts of the viral genome responsible for replication are removed or inactivated, the risk of insertional mutagenesis remains as the CAR sequence is incorporated into the host genome, which could lead to secondary cancers, a concern recently voiced by the FDA [5].

While this risk is low and therefore acceptable in terminally ill patients with limited or no remaining treatment options, it may be less palatable in indications where the patient’s life is not threatened such as autoimmune diseases like systemic lupus erythematosus (SLE) or multiple sclerosis (MS) [6,7,8]. Here, the goal is to deplete the autoreactive B cells that cause the disease and thus reset the immune system without necessarily requiring ongoing surveillance by modified T or other immune cells. The disease burden—percentage of dysfunctional B cells—tends to be much lower compared to in cancer; therefore, theoretically, less-active CAR-T cells are required [9]. And, finally, off-target effects of CAR-T activity such as Immune effector Cell-Associated Neurotoxicity Syndrome (ICANS) may be less likely if the T cells are CAR+ for a short duration in vivo [10,11].

Another advancement in the modified cell therapy field is the application of genome editing approaches such as CRISPR to generate allogeneic material from healthy donors to treat more patients per batch of cells manufactured, which entails knocking out endogenous human leukocyte antigen (HLA) genes (e.g., β2M)—crucial for distinguishing ‘self’ and ‘non-self’—in the T cells by delivering the CRISPR components to the cells during the ex vivo manufacturing process [12]. In both these examples, more transient transgene (CAR, Cas9) expression, such as by using exogenously provided mRNA, is desirable. In light of this, alternative non-viral approaches to CAR-T cell generation have been developed and have proven promising [13,14]. mRNA-based CAR-T represents a potentially safer and cheaper option due to ease of design, production, and scalability [15,16].

The optimal basic structures and sequence of an effective mRNA for therapy that impact RNA integrity, purity and stability, protein yield, and translation efficiency include the promoter, 5′ cap, 5′ untranslated region (UTR), open reading frame, 3′ UTR, stop codon, and poly(A) tail [17,18]. Well-established 5′ and 3′ UTRs of highly expressed and efficiently translated genes continue to be used to enhance expression of exogenous genes of interest. For example, the 5′ UTR of human α-globin or the combination of both a 3′ UTR comprising two sequence elements derived from the Amino-terminal Enhancer of Split (AES) mRNA and the mitochondrial-encoded 12S ribosomal RNA have been used in the vector for COVID-19 mRNA vaccine production by Pfizer and Moderna [19]. Efforts continue to identify new UTR sequences that positively impact mRNA stability and translation efficiency. Using direct analysis of ribosome targeting (DART), more than 30,000 human 5′ UTRs were screened, identifying sequences that mediated more than 200-fold-increased translation [20]. De novo-designed short 5′ UTRs based on secondary structure, length, and GC content have been combined with repeats of different 3′ UTRs to improve translation in vitro and in vivo [21,22]. Recently, functional screens of large numbers of viral sequences have identified positive elements which are bound by ZCCHC-TENT4 enzymes to extend the polyA tail by incorporating adenosine residues and occasionally non-adenosine bases (so called mixed-tailing), thus impeding deadenylation [23,24].

Unmodified RNA can be quite labile and therefore too short-lived, even for the applications described above. Thus, strategies for enhancing the persistence and/or translation of therapeutic mRNA, such as engineering T7 polymerase or chemically modified bases or sugar-phosphate backbone, are being sought [25,26,27].

Since the “rediscovery” of the presence of the N^6^–methyladenosine (m6A) base in mRNA in eukaryotic cells [28,29], there has been a surge of interest in understanding the role of this modification on mRNA function and fate. In mammalian cells, on average, three m6A residues are estimated to occur per RNA transcript. STC-15, an inhibitor of METTL3, the methyltransferase that installs the m6A modification, is currently being used in clinical trials for the treatment of solid tumours [30]. The fates of m6A-containing transcripts are determined by the reader proteins recruited to the modified transcript and tend to be sequence-context-dependent. A commonly accepted mechanism is that m6A acts as a destabilising mark through the CCR4–NOT complex degradation pathway mediated by the YTHDF2 m6A reader [31]. A recently discovered m6A-mediated RNA degradation pathway, termed “CDS m6A decay”, occurs due to ribosome pausing at m6A sites on the coding sequence (CDS). This induces ribosome collision resulting in a unique structural conformation, which, in turn, recruits YTHDF proteins, triggering decay. Thus, the combination of ribosome stalling, codon context, and tRNA modification status determines the susceptibility of a particular m6A-containing transcript to CDS m6A decay [32,33,34,35,36].

We previously showed the positive impact of including m6A consensus DR**A**CH (D = A/G/U, R = A/G, H = A/C/U) sequences in a synthetic 5′ UTR on the yield of human Erythropoietin in CHO cells [37,38]. In addition, we also demonstrated that manipulating the level of m6A reader proteins, YTH-domain family proteins YTHDF1-3, had a significant impact on transgene expression and Lentivirus production in HEK293 cells [39].

In this study, we were interested in understanding the impact, if any, of naturally occurring m6A sites on exogenously delivered in vitro-transcribed mRNA, using chimeric antigen receptor (CAR) expression in T cells as a model system, and whether UTR engineering to include further m6A sites could be a strategy to enhance mRNA stability/translation. The results demonstrate the importance of m6A sites on the expression of CD19-CAR in T cells.

## 2. Results and Discussion

### 2.1. METTL3 Inhibition Reduces Expression of CD19-CAR Protein in IVT mRNA-Transfected T Cells

**Figure 1:**
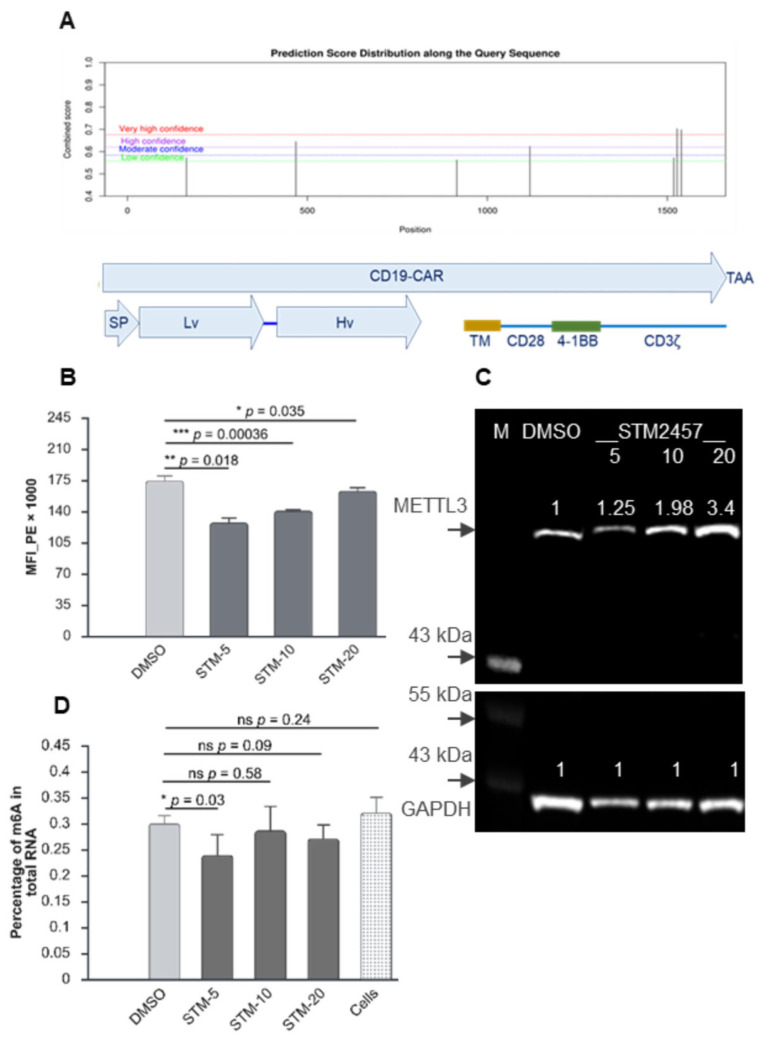
METTL3 inhibition reduces expression of CD19-CAR protein in IVT mRNA-transfected T cells. (**A**) Predicted m6A sites within the CAR gene sequence (top panel). CD19-CAR transcript and encoded regions (bottom panel). Signal peptide (SP). Light-chain variable (Lv). Heavy-chain variable (Hv). Transmembrane (TM). (**B**) Relative Median Fluorescence Intensity (MFI) of activated T cells treated with STM2457 and transiently transfected with CD19-CAR mRNA (n = 3 biological replicates, n = 3 technical replicates) (±SD), * (*p* < 0.05), ** (*p* < 0.01), and *** (*p* < 0.001) (two-sided Student’s *t*-test). (**C**) Western blot analysis of T cells treated with three concentrations of STM2457 (5 µM, 10 µM, and 20 µM) and a control DMSO (**D**), probed with an anti-METTL3 and anti-GAPDH antibodies. The levels of METTL3 protein normalised to GAPDH are shown as numbers above the bands. (**D**) Percentage of m6A in total RNA (n = 2 biological replicates, n = 3 technical replicates) by ELISA (±SD), ns (*p* > 0.05), * (*p* < 0.05), ** (*p* < 0.01), and *** (*p* < 0.001) (two-sided Student’s *t*-test). Graphs created at https://BioRender.com.

A third-generation CAR sequence was used in this work consisting of an extracellular antigen (CD19) recognition domain, transmembrane domain, and CD28 and 4-1BB co-stimulating domains, followed by the CD3ζ signalling domain (Figure 1A) [40,41]. The presence of m6A sites in the coding region of CD19-CAR was predicted using the software program SRAMP (https://www.cuilab.cn/sramp, accessed on 16 January 2022) [42]. This analysis identified four predicted m6A sites with high confidence, two of which had very high confidence scores (Figure 1A). Both very high confidence sites (nt1527 and 1539) were situated in the CD3ζ coding region, while the high-confidence sites were located in the heavy-chain variable (nt468) and CD28 co-stimulatory domain (nt1118), respectively.

To investigate whether m6A methylation had any role to play in CD19-CAR expression, we transfected activated T cells cultured in the presence of an METTL3 methyltransferase-specific inhibitor, STM2457 [43]. The wild-type (wt) CAR transcript used was commercially synthesised (Trilink Biotech, San Diego, CA, USA) and contained a synthetic 5′ UTR, a mouse α-globin 3′ UTR, and a 120nt polyA tail and was capped using CleanCap™ as described by Henderson et al. [17]. Cell surface expression of the CAR protein was significantly reduced at all concentrations of the inhibitor (Figure 1B). This indicated that the CD19-CAR transcript most likely contains sites that are substrates for METTL3. Interestingly, a dose-dependent increase in expression of METTL3 was observed, suggesting that the cellular response to the enzyme’s activity being blocked is to increase its expression, presumably to compensate for METTL3 inhibition (Figure 1C). This response to STM2457 treatment has also been observed in non-small-cell lung cancer A549 and H1975 cells [44]. At the lower concentration of the inhibitor, where METTL3 expression was only slightly increased compared to the DMSO control, there was a small but significant reduction in the levels of cellular m6A (Figure 1D). However, at higher concentrations of the inhibitor, total m6A was not significantly reduced, presumably due to increased METTL3 enzyme expression. While some studies have shown that m6A levels in cancer cell lines are suppressed at higher concentrations of inhibitors despite increased METTL3 expression [44], it has also been reported that primary cells, such as in this study, are less impacted by inhibitor treatment [43].

It is interesting to note that, while METTL3-catalysed methyltransferase activity is mainly restricted to the nucleus, where it depends on the presence of accessory proteins such as METTL14 and WTAP [45], METTL3 is also found in the cytoplasm, where it has been shown to bind mRNAs and promote their translation as well as modifying the RNA genomes of viruses such as hepatitis, Zika, yellow fever, and West Nile [46,47,48]. This would suggest that either the transfected IVT CAR mRNA reaches the nucleus in order to have the m6A modification installed before re-export to the cytoplasm or, more likely, that methylation occurs in the cytoplasm [49].

### 2.2. Mutation of Predicted M6A Sites Reduces the Expression Level of CD19-CAR in T Cells

**Figure 2:**
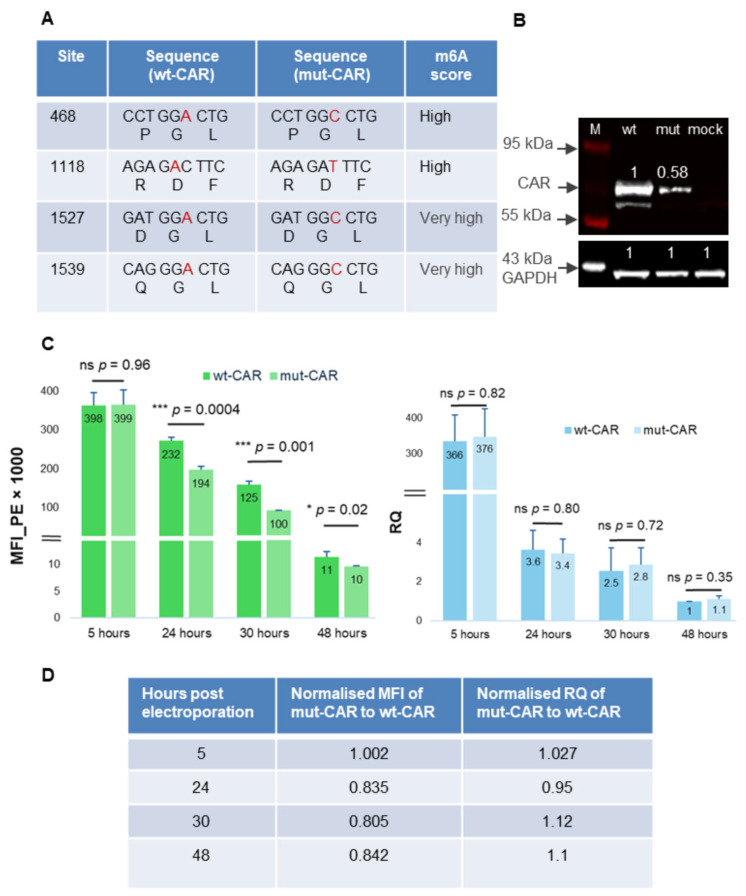
Modification of predicted m6A sites negatively impacts CD19-CAR expression. (**A**) Modification of predicted m6A sites (modified base in red) and the resulting amino acids. (**B**) Western blot analysis of the expression of the wild-type CAR (wt), the mut-CAR (mut), and a mock-transfected control in T cells 24 h post electroporation. The levels of CD19-CAR protein normalised to GAPDH are shown as numbers above the bands. (**C**) Median Fluorescence Intensity (surface protein) and relative mRNA quantification (RQ) of wt-CAR and mut-CAR at four time points post electroporation in activated T cells (n = 2 biological replicates; n = 3 technical replicates) ±SD, ns (*p* > 0.05); * (*p* < 0.05); ** (*p* < 0.01), and *** (*p* < 0.001) (two-sided Student’s *t*-test). (**D**) Summary of normalised MFI and RQ at different time points post electroporation. Figure created at https://BioRender.com.

The consensus motif for m6A is DRACH (D = A, G or U; R = A/G; H = A, C or U). However, DRACH sequences appear frequently in genes and only a small number of them turn out to be substrates for methylation. Instead, it is the context of the DRACH motif in terms of secondary structure that dictates the likelihood of m6A occurring [28,50]. In the CD19-CAR sequence, three of the four in silico predicted sites were GGACT—the canonical DRACH—and the fourth was AGACT. Synonymous mutations were generated at these sites to either remove the A or disrupt the DRACH consensus—but maintain the encoded amino acids—and the mutated version (mut-CAR) was generated (TriLink Biotech, San Diego, CA, USA) using the same template vector as for the wild type (wt-CAR) (Figure 2A).

The IVT mRNAs were transfected into activated T cells by electroporation, and subsequent CAR protein expression was monitored. Western blot analysis of protein lysates 24 h post electroporation confirmed that whole CAR protein levels were diminished in T cells transfected with the mutated mRNA compared to the wt-CAR (Figure 2B).

Surface CAR expression was monitored at various timepoints after electroporation of activated T cells. At 5 h post electroporation, cells displayed similar levels of CAR expression; however, at 24, 30, and 48 h, the mut-CAR surface expression was significantly lower (~15–20%) than wt-CAR cells (Figure 2C,D). This observation was replicated across different donor-derived T cells with an apparent donor-dependent variation in magnitude of the difference and >50% reduction in mut-CAR expression in some cases (Figure S1A).

In order to establish whether this effect was a consequence of mRNA stability, the amount of mRNA at each timepoint was monitored and revealed an almost identical rapid drop in RNA levels (~100-fold) in both wt-CAR and mut-CAR cells between 5 and 24 h (Figure 2C,D). Both transcripts continued to degrade at a similar rate over the following 24 h, implying that mRNA stability was not the reason for the observed reduction in surface protein and instead reduced translational efficiency in the absence of the m6A modifications. Finally, as most RNA therapies typically incorporate N^1^-methyl-pseudouridine (Ψ) in order to reduce the activation of RNA-sensing cellular mechanisms, we also used wt and mut Ψ versions of the CAR transcripts (generated by TriLink Biotech, San Diego, CA, USA) and observed a similar pattern to the uridine-containing transcripts, i.e., reduced surface CAR expression from the mutant CAR over time (Figure S1B).

It has been well established that the presence of an m6A in the coding sequence is often associated with reduced translation elongation, whereby the modified base causes the ribosome to pause. However, this effect tends to be both context- and m6A-reader-dependent [51,52]. For example, members of the m6A reader YTH-domain-binding family, which are known to induce degradation of m6A-containing mRNAs [53], have also been reported to be involved in enhancing translation [54,55,56]. Furthermore, several recent studies reported Insulin-like growth factor m6A-binding proteins' (IGF2BP1-3) roles in mRNA stability and translation enhancement [57,58,59,60,61].

Finally, we generated an extreme version of the CAR mRNA with all except five ‘RAC’ sequences (core of motif) modified while maintaining the wild-type amino acid sequence (RACmod-CAR); five could not be modified without disrupting the protein sequence (Figure S1C). It should be noted that the RACmod-CAR and the control in this comparison had a different 5′ UTR (min-CAR, used in a later section) than the wt-CAR but with identical ORF sequence; nonetheless, cell surface expression of RACmod-CAR was once again lower than the unmodified CD19-CAR (Figure S1C). These findings further confirmed a role for m6A in influencing the expression of a CD19-CAR incorporating the identified DRACH motifs.

Interestingly, a survey of publicly available sequences of CD19-CARs revealed that homology at the nucleotide level can vary considerably. This can be seen when comparing the nucleotide sequences of the third-generation CAR transcript used in this study to two second-generation CD19-CAR vectors lacking the 4-1BB domain—clone TRAC-CD19-CAR [62] and GenBank Accession number HM852952—where the amino acid sequences of the antigen recognition, CD28 and CD3-ζ domains, apart from one amino acid, are identical (Figure S2), whereas this drops to ~75% at the DNA level for the antigen recognition domain and 82–89% for the CD28 and CD3-ζ domains (Figure S3C). This observation has implications for transgene sequence design and optimisation as it means the fate of any given transcript could be more, or less, susceptible to m6A-dependent post-transcriptional control mechanisms depending on the number of functional DRACH sites present. However, an in silico prediction of m6A sites in these three CD19-CARs using deepSRAMP [63]—an updated version which became available recently and detects more sites with different probability scores, in some cases, than the original SRAMP program—revealed quite diverse m6A predictions (Figure S3A,B), suggesting that accurate prediction of an m6A modification at a given site, in particular those which are not in the software training database, is still challenging. Therefore, these programs should be treated as guides only, requiring experimental validation.

### 2.3. Confirmation of the Presence of m6A-Modified Bases at the Predicted Sites in the Coding Sequence of CD19-CAR

Having established strong phenotypic evidence of m6A impacting the fate of the CAR mRNA, we next sought to directly confirm the presence or absence of methylated adenosine bases at the various sites. Direct long-read RNA sequencing (Oxford Nanopore™) combined with dedicated software algorithms facilitated modified base detection at single-base resolution [64]. In this experiment, a CAR-T sample was also generated using lentiviral (LV) transduction. This was included to establish whether the provenance of an mRNA, i.e., transcribed in and exported from the nucleus or delivered exogenously to the cytoplasm, impacts its methylation pattern. To this end, total RNA was extracted from LV-CD19-CAR-transduced (LV-CAR) or IVT-CD19-CAR-mRNA-electroporated (IVT-CAR) activated T cells. As a non-methylated control, total RNA was extracted from un-transfected activated T cells and IVT-CD19-CAR mRNA was spiked into the sample (UT-IVT-CAR).

**Figure 3:**
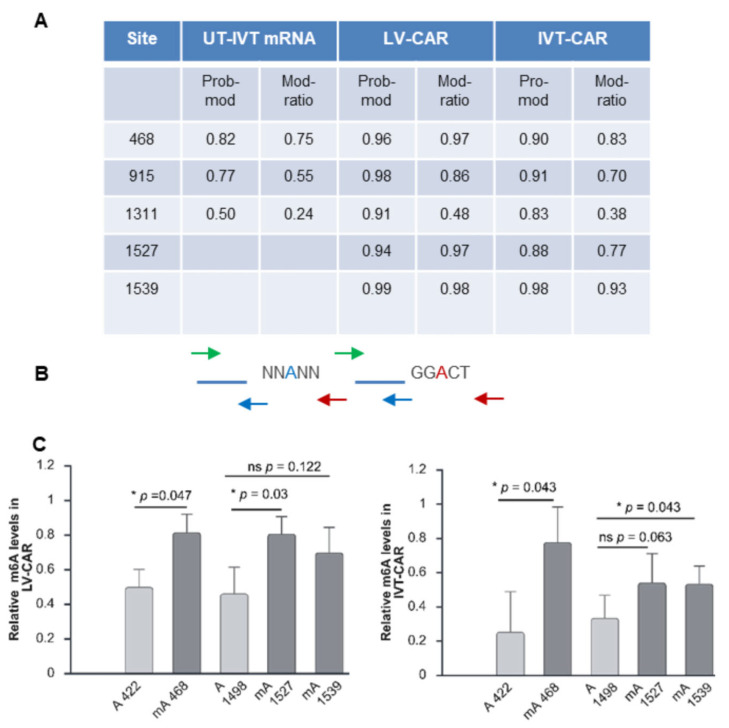
Confirmation of the presence of m6A-modified bases at the predicted sites in the coding sequence of CD19-CAR. (**A**) Probability of modification (Prob-mod) and Modification Ratio (Mod-ratio) at the predicted sites on CD19-CAR by direct nanopore sequencing (n = 1 biological replicate). (**B**) Location of primers used in m6A-qPCR; reverse primers (red arrows) adjacent to an m6A site (red A) or a non-modified A (blue A). Non-adjacent reverse primers: blue arrows; forward primers: green arrows. (**C**) Relative m6A levels at nt468, nt1527, and nt1539 compared to nearby control A sites (A422 and A1498) in LV-CAR (left panel) and IVT-CAR (right panel) transcripts (n = 2 biological replicates)*;* n = 3 technical replicates (±SD), ns (*p* > 0.05); * (*p* < 0.05); ** (*p* < 0.01), and *** (*p* < 0.001) (two-sided Student’s *t*-test). Graphs and figure created at https://BioRender.com.

Analysis of the RNAseq dataset allowed for the identification of individual m6A bases, where a probability score > 0.9 indicates a positive hit. The percentage of modified to unmodified bases at a given base position was also captured. In the LV-CAR-transduced sample, the probability scores for three of the four in silico predicted sites supported the presence of an m6A base (Figure 3A). Furthermore, two other DRACH sites were identified as being m6A-modified—one was predicted by the SRAMP software (nt915) at low confidence—which would not otherwise have been anticipated. Of the five m6A sites found in the LV-CAR, four were modified in the majority (86–98%) of transcripts sequenced, with one (nt1311) containing m6A in approximately half of the transcripts. In comparison, the IVT-CAR-derived transcripts were only found to contain m6A at three sites (nt468, 915, and 1539), although nt1527 was only just below the probability cut-off at 0.88. The percentage of transcripts containing the modification at each site was also generally lower than those in the LV-CAR sample. One of the predicted sites (nt1118) did not pass the threshold in either the IVT-CAR or LV-CAR samples (Figure S4). As expected, no adenosine bases in the UT-IVT-CAR negative control CAR mRNA sample reached the probability threshold, although it should be noted that data were missing for four DRACH sites at the 3′end (Figure S4), most likely due to a sequencing coverage issue, as reported by other groups (https://github.com/GoekeLab/m6anet/issues/133, accessed on 3 September 2025).

As the long-read sequencing was performed as an initial screen of the CAR mRNAs, we employed m6A-sensitive RT-PCR as an orthogonal method of m6A detection. This method takes advantage of the reduced capacity of the BstI reverse transcriptase from *B. stearothermophilus* to retrotranscribe m6A residues compared to that of the Moloney Murine Leukemia Virus (MMLV) enzyme [65]. Utilising this approach, the relative m6A levels at three of the in silico predicted sites with high RNAseq scores from the LV-CAR and IVT-CAR samples were determined. As shown in Figure 3C, the relative m6A levels at nt468, 1527, and 1539 were higher than those of the control A sites in both transduced and transfected T cells. This was consistent with the direct RNAseq data, although nt1539 in the LV-CAR samples and nt1527 in the IVT-CAR samples were not statistically significant. There were no significant modifications detected in negative control UT cells spiked with IVT-CD19-CAR mRNA (Figure S5).

### 2.4. Impact of Additional 5′ and 3′ UTR Sequences Including Those with Consensus DRACH Motifs on CD19-CAR Expression

The consequences of an m6A being present within any given transcript can be difficult to anticipate as it may be transcript-dependent, sequence-context-dependent (secondary structure), and dependent on what proteins engage with the m6A motif. However, from studies so far, there seems to be some general observations linking the location of the m6A site in the transcript with the most likely impact on mRNA fate. For example, m6A in the 5′ UTR has been demonstrated to promote cap-independent translation by binding eiF3, particularly under conditions of stress [66]. On the other hand, m6A in the 3′ UTR can have a major impact on mRNA stability by recruiting reader proteins, such as YTHDF1-3, which, in turn, attract the CCR4-NOT1 deadenylation complex [67,68]. It can also influence translation as well as mRNA cellular localisation [69]. The presence of an m6A in the coding sequence has recently been found to be associated with mRNA degradation via CDS-m6A decay (CMD), which is fast- and translation-rate-dependent [32,34]; however, transcripts with m6A in the CDS have also been shown to be more stable and translationally enhanced [57]. Thus, the impact of the presence of m6A seems to be highly context dependent.

**Figure 4:**
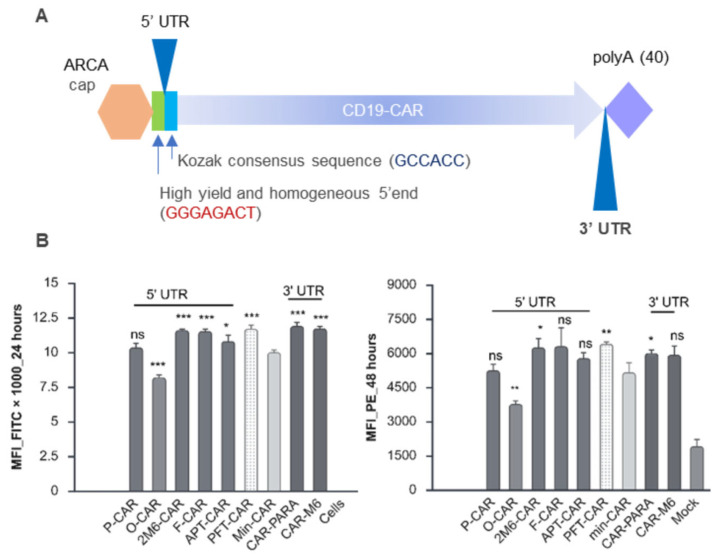
Impact of various 5′ and 3′ UTRs on CD19-CAR expression. (**A**) Features of CD19-CAR mRNA with a minimal 5′ UTR (min-CAR). (**B**) Relative Median Fluorescence Intensity (MFI) of activated T cells 24 h after electroporation with CD19-CAR mRNAs (left panel) and 48 h post electroporation (right panel); n = 3 biological replicates, n = 3 technical replicates (±SD); ns (*p* > 0.05); * (*p* < 0.05); ** (*p* < 0.01), and *** (*p* < 0.001) (two-sided Student’s *t*-test); *p* values at 24 h: *p* = 0.16 (P-CAR); *p* = 0.00017 (O-CAR); *p* = 0.00010 (2M6-CAR); *p* = 0.00025 (F-CAR); *p* = 0.036 (APT-CAR); *p* = 0.0006 (PFT-CAR); *p* = 0.0003 (CAR-PARA); *p* = 0.0001 (CAR-M6); *p* values at 48 h: *p* = 0.77 (P-CAR); *p* = 0.005 (O-CAR); *p* = 0.029 (2M6-CAR); *p* = 0.096 (F-CAR); *p* = 0.090 (APT-CAR); *p* = 0.007 (PFT-CAR); *p* = 0.029 (CAR-PARA); *p* = 0.078 (CAR-M6). Graphs and figure created at https://BioRender.com.

Encouraged by the findings that implicated a positive role for m6A in CD19-CAR expression, we were interested to determine whether we could further improve CAR protein expression by engineering the 5′ and 3′ UTRs flanking the CAR coding sequence. To this end, we designed a minimal CD19-CAR (min-CAR) comprising a short synthetic 5′ UTR that consisted of a basic T7 promoter, a short sequence after the TATA element of the T7 promoter required for high yield, and homogenous 5′ ends during mRNA in vitro transcription (GGGAGACT) upstream of a Kozak consensus sequence (GCCACC) that has been previously described (Figure 4A) [21]. We then chose several recently reported 5′ UTR and 3′ UTR sequences as well as some de novo-designed sequences, some of which incorporated potential m6A sites and some not, to test their effect on CAR expression.

We tested a short 5′ UTR (2M6-CAR) containing two m6A consensus sequences, which had been previously reported to increase translation of cellular mRNAs and recombinant transgenes [37]; an unstructured short sequence that enhanced the translation of a Nanoluc reporter in circular RNA (O-CAR) [70]; an eIF4G aptamer that recruits the eukaryotic initiation factor 4E (eIF4E) protein to the 5′ end of transfected IVT mRNA, which was reported to enhance reporter gene expression by up to tenfold in various cell types (APT-CAR) [71]; and a synthetic 5′ UTR selected from a library of 280,000 random sequences, which increased GFP expression by threefold in Hela cells (F-CAR) [72,73]. We included a 5′ UTR derived from the human alpha-globin gene and used in the Pfizer SARS-CoV2 mRNA vaccine (WHO-Pfizer sequence 11889), which was predicted to contain one m6A site, as a control (P-CAR). We also examined the impact of incorporating a consensus m6A site in the 3′ UTR (CAR-M6), which has been implicated in enhancing translation by recruiting YTHDF1 or METTL3, which, in turn, recruits eIF3 to the 5′ end of the mRNA, promoting further interaction with other translation initiation factors including eIF4G [46,74]. Another 3′ UTR sequence tested was a 16-mer motif from the 3′ UTR of variant surface proteins (VSGs) in *Trypanosoma brucei*, a unicellular eukaryotic parasite that causes lethal disease, including sleeping sickness, in humans (CAR-PARA). This motif is required for inclusion of m6A in the poly(A) tail, which, when removed, results in poly(A) tails lacking m6A, rapid deadenylation, and subsequent mRNA degradation [75]. Finally, we included another control comprising the CAR coding sequence flanked by both the 5′ and 3′ UTR sequences used in the Pfizer SARS-CoV2 mRNA vaccine (PFT-CAR). Sequences and features of the 5′ UTR and 3′ UTRs are summarised in Table 1.

All the constructs were synthesised and cloned in pUC57 (GenScript), and IVT mRNAs were generated using an ARCA kit. The IVT mRNAs were delivered into activated T cells by electroporation and the cell surface expression of CAR protein was monitored by flow cytometry. The day after transfection, most of the tested 5′ and 3′ UTR sequences—2M6-CAR, APT-CAR, PFT-CAR, CAR-PARA, and CAR-M6—resulted in a modest but significant positive impact on CAR expression, typically a 15–20% increase compared to the min-CAR control (Figure 4). The P-CAR had no impact, which was consistent with what has been reported previously in A549 cells [21]. The O-CAR sequence, despite enhancing circRNA reporter gene expression in Hela, reduced CAR expression by nearly 20%. This transcript had very weak predicted secondary structure based on minimum fold energy (MFE) (Table 1); however, weak secondary structure does not always imply high gene expression [19]. In contrast to the neutral impact of the 5′ UTR of human α-globin (P-CAR), the PFT-CAR, which also contained a 3′ UTR comprising two sequence elements derived from the Amino-terminal Enhancer of Split (AES) mRNA and the mitochondrial-encoded 12S ribosomal RNA, increased CAR expression by ~17% at 24 h. This sequence has been reported to confer mRNA stability and high total protein expression [76]. Clearly, for this construct, the positive impact can be attributed to the 3′ UTR sequence. A similar profile of CAR expression across the panel of constructs was also observed after 48 h. A summary of the results is shown in Table 1.

### 2.5. A 3′ UTR Containing Tandem Repeats of m6A Motifs and Vsg 16-mer Sites Positively Impacts CD19-CAR Expression Compared to 3′UTRs with Strong Secondary Structures

As both the 16-mer from the vsg 3′UTR (CAR-PARA) and a single m6A consensus sequence (CAR-M6) in the 3′ UTR of CD19-CAR conferred a positive effect on the expression of the target mRNA, we were interested to see whether repeating these motifs (CAR-2M6-2v16 and CAR-2M6L-2v16) and combining them with other extensively used 3′ UTR elements would enhance the effect. For example, the woodchuck hepatitis posttranscriptional regulatory element (WPRE) and the SV40 early/late polyadenylation signal are commonly used for efficient transgene expression in mammalian cells. The WPRE is quite long; however, a 247nt shorter version has been shown to be as effective as the standard 600nt version [77]. In addition, this short WPRE, in combination with a tandem repeat of SV40 upstream late enhancer followed by an SV40 late polyadenylation signal, resulted in high eGFP expression in HEK293 cells [78]. We tested this sequence alone (CAR-W3SL) or in combination with 2m6A-v16mer motif downstream (CAR-W3SL2M6v16). In addition, we tested a combination of 2 × m6-v16mer and the SV40 late polyadenylation signal (SL) (CAR-2M6v16SL). Finally, we tested a construct (2M6-CAR-2M6v16SL70) which combined the m6A motif in the 5′UTR (2M6-CAR) and a 3′UTR consisting of 2m6-v16mer-SL plus a 70nt poly(A), instead of the standard 40nt poly(A), as well as an AT-rich sequence (ATTTA), which is bound by the human antigen R (HuR) protein and contributes to increased stability and longer transcript half-life [79].

**Figure 5:**
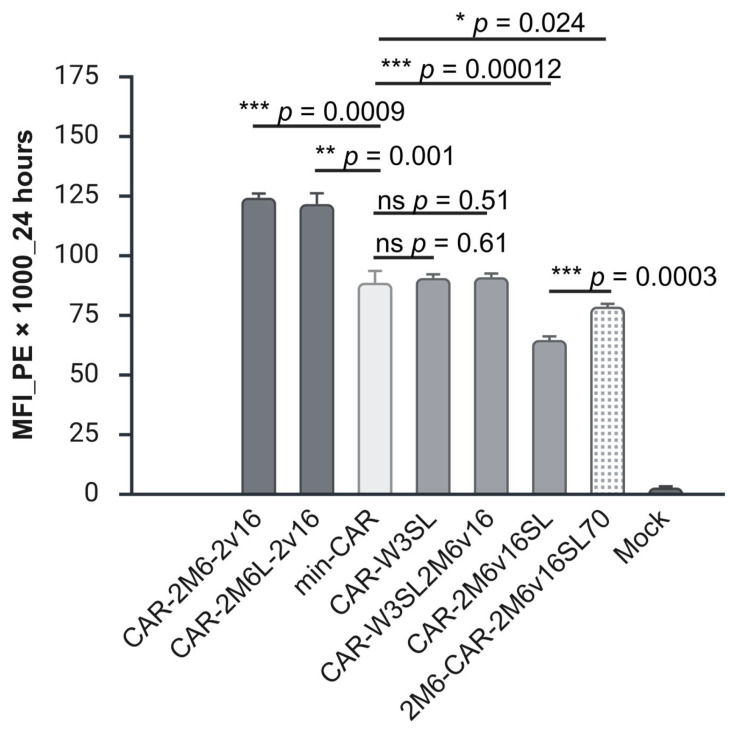
Impact of 3′ UTR containing repeated motifs or high secondary structure on CD19-CAR expression. Relative Median Fluorescence Intensity (MFI) of activated T cells transiently expressing CD19-CAR 24 h post electroporation; n = 3 biological replicates, n = 3 technical replicates; (*±*SD); ns (*p* > 0.05), * *p* < 0.05; ** *p* < 0.01, *** *p* < 0.001 (Student’s *t*-test). Graph created at https://BioRender.com.

Transfected T cells were analysed 24 h post electroporation; however, only the samples with a combination of 2 × m6A and v16-mer (2M6-2v16)—of which there were two, with different length spacers between them (constructs CAR-2M6-2v16 and CAR-2M6L-1v16)—showed a positive impact with a ~40% increase in CAR protein (Figure 5 and Table 2). The 3′ UTR with the short WPRE and SV40 late poly A signal sequence with (construct CAR-W3SL2M6v16) or without 2M6-2v16 (construct CAR-W3SL) did not improve expression of CD19-CAR. On the other hand, omitting the short WPRE sequence reduced expression of CD19-CAR by nearly 25% (construct CAR-2M6v16SL); however, the negative effect of excluding the short WPRE sequence seems to be compensated either by the presence of the 5′ UTR containing 2M6A sites or the presence of the AT-rich sequence and longer polyA (construct 2M6-CAR-2M6v16SL70), as protein expression increased by 15%.

Notably, all the 3′ UTR sequences containing WPRE or SV40 harbour miR-4719-binding sites (prediction score: 97–100%), which might be a contributing factor to the observed result [80]. However, despite the prediction of high-to-very-high confidence scores for m6A methylation, m6A-qPCR did not provide any evidence of methylation at these sites (Table 2). It was not possible to verify the two engineered m6A sites in the 5′ UTR of the construct 2M6-CAR-2M6v16SL70 by m6A-qPCR as the sites were too near the transcriptional start site to design suitable primers. Therefore, it seems that, for the CAR-2M6-2v16 and CAR-2M6L-2v16 constructs at least, the increase in CAR expression was m6A-independent.

### 2.6. Consensus m6A Sites in the 3′ UTR Are Methylated in a Certain Sequence Context

**Figure 6:**
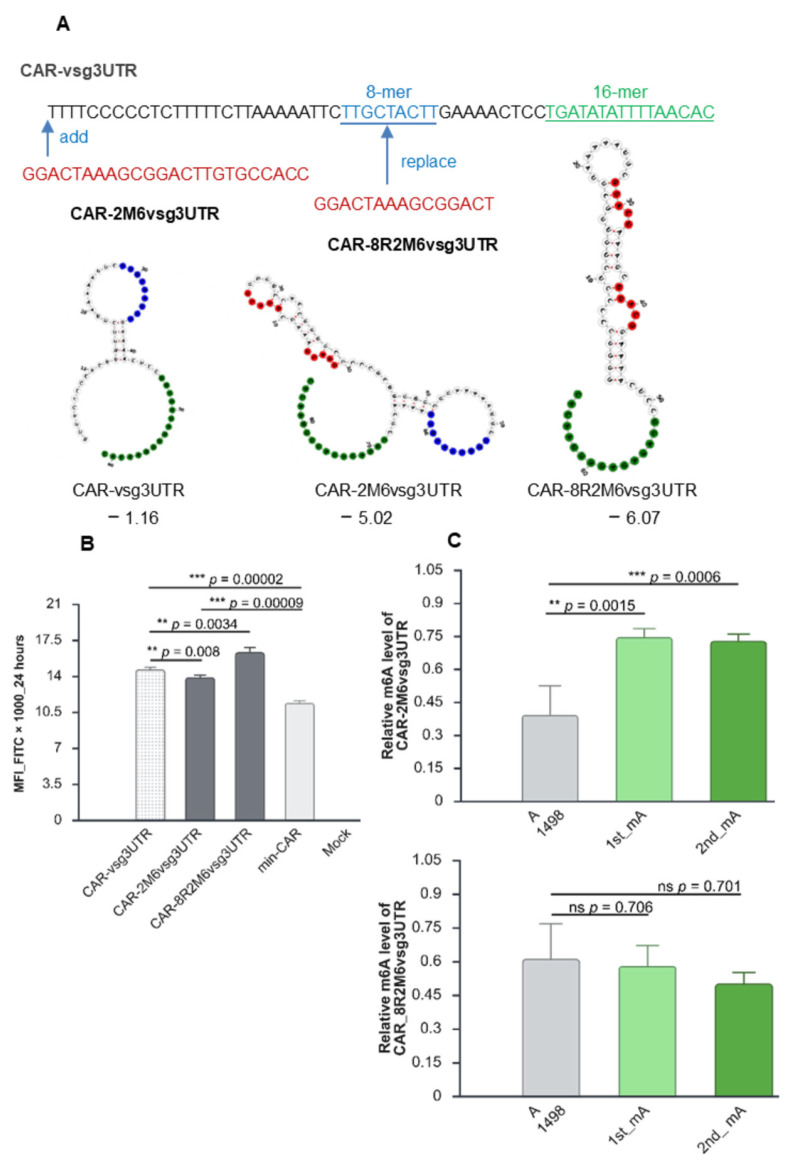
Consensus m6A sites in the 3′ UTR are methylated in a certain sequence context. (**A**) Full-length sequence of the 3′ UTR of variant surface genes (VSGs) and two versions with m6A consensus sites and their secondary structures. (**B**) Relative Median Fluorescence Intensity (MFI) of activated T cells 24 h after electroporation (n = 2 biological replicates, n = 3 technical replicates). (**C**) m6A levels at the first and second consensus m6A sites relative to nearby control (A1498) site in CAR-2M6vsg3UTR (top panel) and CAR-8R2M6vsg3UTR (bottom panel)*;* n = 2 biological replicates; n = 3 technical replicates (±SD); ns (*p* > 0.05); * (*p* < 0.05); ** (*p* < 0.01), and *** (*p* < 0.001) (two-sided Student’s *t*-test); first (1st); second (2nd); red letters: consensus m6A sites; blue letters: vsg-8-mer; green letters: vsg-16-mer. Graphs and figure created at https://BioRender.com.

So far, none of the consensus DRACH sites engineered into the 3′ UTR of CD19-CAR were found to be subjected to m6A methylation. The vsg-16-mer in the 3′ UTR positively impacted CAR expression (CAR-PARA), similar to that of construct CAR-M6; however, because this appeared to be independent of m6A, we speculated that secondary structure could be responsible. The vsg-16-mer is a conserved region among different parasite strains and is essential for the stability and functional levels of expression of vsg genes in the parasite [81]. The entire vsg 3′ UTR is 61 nucleotides with no strong secondary structure (Figure 6A). In addition to the conserved 16-mer, an 8-mer in the 3′ UTR of vsg is also conserved among different parasite strains [82]; however, is not associated with mRNA abundance or stability of vsg transcripts. We made two constructs both incorporating two m6A consensus sites, but with slightly different predicted secondary structures, in addition to a full-length vsg-3′UTR (CAR-vsg3UTR) construct as a control. The first construct contained two m6A consensus sites added upstream of the vsg 3′ UTR (CAR-2M6vsg3UTR) and the second with two m6A consensus sites replacing the 8-mer of the vsg-3′ UTR (CAR-8R2M6vsg3UTR) (Figure 6A, Table 3). All three constructs conferred significantly increased surface expression of CAR compared with the min-CAR—21% (CAR-2M6vsg3UTR), 28% (CAR-vsg3UTR), and 43% (CAR-8R2M6vsg3UTR) (Figure 6B). Both m6A sites engineered in the constructs CAR-2M6vsg3UTR and CAR-8R2M6vsg3UTR were predicted to be targeted for m6A methylation (SRAMP) and both sites were at similar positions in the junction of stem–loop structures. However, only the two sites in the construct CAR-2M6vsg3UTR were verified to be methylated by m6A-qPCR, while those in CAR-8R2M6vsg3UTR were not (Figure 6C). The untreated (UT) control with spiked CAR-2M6vsg3UTR IVT mRNA showed no methylation by m6A-qPCR, as expected (Figure S6). These data seem to imply that the presence of m6A in the context of the CAR-2M6vsg3UTR reduced the positive impact of the full-length vsg-3′ UTR (construct CAR-vsg3UTR) on the expression of CAR. On the other hand, the replacement of the vsg 8-mer increased CAR expression by ~15% compared to the full-length vsg-3′ UTR. It remains to be determined whether the positive effects on CAR expression from CAR-vsg3UTR and CAR-8R2M6vsg3UTR share similar mechanisms, as has been found in the parasite, which involves preventing mRNA deadenylation [75]. It would also be interesting to establish whether there is a different cohort of reader proteins binding to m6A sites in the coding and in the 3′ UTR of the construct CAR-2M6vsg3UTR.

## 3. Materials and Methods

### 3.1. Cell Lines, Lentiviruses, and Reagents

Pan-T cells were sourced from Stem Cell Technologies (Vancouver, BC, Canada), and Charles River Laboratories (Cambridge, MA, USA). Anti-CD19-CAR Lentiviruses were produced by Sirion Biotech (Graefelfing, Germany). Details of all reagents are in Supplemental Table S1.

### 3.2. In Vitro mRNA Transcription

The coding sequences of anti-CD19 CAR variants, including the wild-type anti-CD19-CAR (min-CAR), were synthesised by GenScript (Rijswijk, Netherlands) unless otherwise stated. The plasmids were linearised using a restriction enzyme downstream of the poly(A) tail, cleaned, and concentrated using the DNA Clean & Concentrator^®^-25 kit (Cat# D4033, Zymo Research, Irvine, CA, USA). In vitro-transcribed (IVT) mRNAs were produced with ARCA cap using High-Yield T7 ARCA mRNA Synthesis Kit (Cat# RNT-102-L, Jena Biosciences, Thuringia, Germany) and yeast inorganic pyrophosphatase (Cat# M2403S, NEB, Ipswich, MA, USA) at a final concentration of 0.002U/ul. The resulting mRNAs were then treated with DNase I (Cat# M0303S, NEB) and cleaned using Monarch RNA cleanup kit preps (Cat# T2050S, NEB). DNA-free mRNA quality was checked by Nanodrop and Agilent TapeStation (Santa Clara, CA, USA). RNA concentration was measured using an Invitrogen™ Qubit™ RNA High Sensitivity kit (Cat# Q32852, Invitrogen, Carlsbad, CA, USA) and a Qubit fluorometer.

### 3.3. In Vitro Electroporation

Freshly thawed Pan-T cells were activated for three days in serum-free TexMACS™ Medium (Cat# 130-097-196, Miltenyi, Bergisch Gladbach, Germany) supplemented with human IL-7 (155 U/mL) and IL-15 (290 U/mL) using T Cell TransAct™ (Cat# 130-111-160, Miltenyi), according to the manual provided by the supplier (day 0). Activated Pan-T cells were verified using FITC-anti-CD3 and anti-CD25 antibodies (Miltenyi). One million activated T cells were electroporated with 3.3 μg of IVT mRNA using P3 Primary Cell 4D-Nucleofector^TM^ X kit (Cat# V4XP-3032, Lonza, Basel, Switzerland) and pulse code EO-115 on a 4D-NucleofectorTM X Unit. Each transfection was repeated two or three times (biological replicates), each time in 3 wells (technical replicates). Cells collected 24 or 48 h post electroporation for RNA and protein analysis were washed with phosphate-buffered saline (PBS), re-suspended in TRIzol solution (for RNA prep), or stored at −70 °C for protein lysate preparation.

### 3.4. Lentiviral Transduction

Five million 3-day-post-activation T cells (day 3) were transduced using Lentivirus at Multiplicity of Infection (MOI) = 30 (day 3). Transduced T cells were expanded further in TexMACS™ Medium supplemented with human IL-7 (155 U/mL) and IL-15 (290 U/mL). Transduced T cells were then collected for RNA and protein analysis on day 9. Each transduction was performed in 3 biological replicates and 3 technical replicates.

### 3.5. CAR Detection by Flow Cytometry

Anti-CD19-CAR expression in transfected or transduced cells was monitored by staining with fluorophore-conjugated antibody (CD19 CAR Detection reagent, human (Cat# 130-129-550), biotin and PE-Biotin, REAfinity™ (Cat# 130-111-068) (Miltenyi), or FITC-Labelled Monoclonal Anti-FMC63 Antibody (Cat# FM3-FY45, ACROBiosytems, Newark, DE, USA) according to the protocol supplied. At least 100,000 cells were used for staining. Dead cells were excluded using 7-AAD (Cat# 130-111-568, Miltenyi) or SYTOX red (Cat# S11380, Thermofisher Scientific, Waltham, MA, USA) in combination with PE-Biotin (MFI-PE) or FITC label (MFI-FITC), respectively. Flow cytometry analysis was performed on a BD Accuri™ C6 Plus system (BD Biosciences, Paramus, NJ, USA). Untreated activated T cells were stained the same way and were used for gating. The stained cells were first gated for single cells using scatter blot (forward scatter height (FSC-H) versus forward scatter area (FSC-A) and then for viable cells via 7-AAD or SYTOX red negative signal. All results were expressed as mean ± SD. A two-sided Student *t*-test value of ≤0.05 was considered statistically significant.

### 3.6. M6A-qPCR

Relative m6A levels were determined using the M6A- qPCR method developed by Castellanos-Rubio et al. [67]. Briefly, 150 ng DNA-free total RNA isolated using Direct-zol™ RNA MiniPrep kit (Zymo Research, Orange, CA, USA) was reverse-transcribed (RT) with Bst 2.0 (NEB) or Superscript IV (Invitrogen) and a reverse primer using the protocol specified in the manual. For qPCR, 1.5 μL of the resulting RT product was used in a quantitative PCR reaction using Applied Biosystems™ PowerUp™ SYBR™ Green Master Mix (Thermofisher Scientific) or SYBR^TM^ Fast SYBR Green Master Mix (Thermo Fisher Scientific) on a Quanstudio 3 or a Quanstudio 5 instrument. Primers (Supplement Table S1) were supplied by Integrated DNA Technologies (IDT, Coralville, IA, USA). For the control, in vitro CD19-CAR mRNA was spiked in at a final concentration of 0.0003% of total RNA of untreated T cells (equivalent to that of β-actin) for the RT reaction.

### 3.7. Western Blots

Cell pellets were resuspended and lysed in RIPA buffer (Thermo Scientific) containing Halt^TM^ protease inhibitor cocktail at 4 °C for 1 h with gentle rotation. Cellular debris was removed by centrifugation at 14,324× *g* for 20 min, and the supernatant was collected. The resulting protein samples were heated at 70 °C for 10 min in the presence of 1x SDS loading buffer. Proteins were separated on a precast 4–12% Bis-Tris Plus gel in Bolt™ MOPS SDS running buffer (Invitrogen). The separated proteins were blotted on a 0.45 µm nitrocellulose membrane (Amersham) using Thermo Scientific Pierce Power Blotter in Pierce 1 step transfer buffer. Primary antibodies for CAR (Cat# 12837-2-AP, ProteinTech, Chicago, IL, USA); for METTL3 (Cat# A301-567A-T, Abcam Cambridge, UK); and for of GAPDH (Cat# 60004-1-IG, Proteintech) were used at the recommended dilution. IRDye 680RD and IRDye^®^ 800CW secondary antibodies were from Licorbio (Lincoln, NE, USA). Blots were scanned and quantitatively analysed using the Odyssey ^®^ Fc Imaging System.

### 3.8. qRTPCR

cDNA was prepared using a High-Capacity cDNA Reverse Transcription Kit following the manufacturer’s protocol. qPCR was performed using a FAST SYBR kit with three technical replicates and analysed on a Quanstudio 3 or 5 (Thermofisher). Relative gene expression was calculated using the 2^−ΔΔCt^ method with GAPDH as a reference gene. Primers used are listed in Table S1.

### 3.9. STM2457 Treatment

STM2457 (Cat# HY-134836, MedChemExpress, MedChemExpress, NJ, USA New Jersey, USA) 10 mM stock solution was prepared in DMSO following the instructions of the manufacturer and stored at −80 °C in small aliquots. A working solution of STM2457 was prepared in TexMACS™ medium before use. Activated T cells were incubated overnight in different concentrations of STM2457 or a DMSO control before being transfected with IVT mRNAs. Cells were analysed 24 h post electroporation.

### 3.10. Total RNA Preparation for Nanopore RNA Direct Sequencing

DNA-free total RNA was prepared using the Direct-zol™ RNA MiniPrep kit (Zymo Research). The RNA quality and concentration were checked on a Tape Station (RNA integrity from 9.5 to 10) and Invitrogen™ Qubit™ RNA High Sensitivity kit and a Qubit fluorometer. For RNA from untreated T cells, CD19-CAR mRNA (synthesised by TriLink) was added to a final concentration of 0.0003% total RNA (equivalent to that of β-actin).

### 3.11. M6A Mapping by Nanopore Direct RNA Sequencing

Nanopore direct RNA sequencing was performed by OHMX-Bio (Ghent, Netherlands). At least 57 μg total RNA was used for each run. The Direct RNA Sequencing Kit (Cat# SQK-RNA004, Oxford Nanopore Technologies, Oxford, UK) was used. Briefly, total RNA samples were ligated to the RTA adapter with the T4 DNA Ligase (NEB) and then reverse-transcribed with Superscript III Reverse Transcriptase and dNTPs (Thermo Fisher). RNA-cDNA hybrids were purified with Agencourt RNAClean XP beads (Beckman Coulter, Brea, CA, USA) and ligated to the sequencing RNA Ligation Adapter (RLA) using T4 DNA Ligase. Following final purification with RNAClean XP beads, the concentration of the library was determined on the Qubit Fluorometer with the Qubit DNA HS Assay kit (Thermo Fisher).

All three libraries were sequenced individually on the P2Solo using FLO-PRO004RA flow cells (Oxford Nanopore Technologies). The flow cell was first primed with flow cell priming mix consisting of RNA Flush Tether (RFT) and Flow Cell Flush (FCF) from the Direct RNA Sequencing Kit (SQK-RNA004, Oxford Nanopore Technologies). Each RNA library was then mixed with Library Solution (LIS) and Sequencing Buffer (SB). Subsequently, 32 μL of this mixture was loaded onto the flow cell. Sequencing lasted for a total of 72 h.

Dorado base calling was completed using the high-accuracy model in MinKNOW. M6A sites were identified using m6Anet software (version 2.1.0), with Ensembl release 106 was used for the Homo sapiens (human) data (assembly GRCh38) and CD19-CAR sequence added under the name ‘sequence_NIBRT’ as reference. The developers of m6Anet recommend using a threshold of 0.9 in the ‘probability_modified’ column to select m6A sites. The software m6Anet (v2.1.0) used zero-based indexing to report these positions. This means that the first base position starts at 0.

### 3.12. Sequence Analysis

The following programs were used for sequence analysis: Clustal Omega (https://www.ebi.ac.uk/jdispatcher/msa/clustalo, accessed on 3 September 2025) [83] for protein and DNA multiple sequence alignment; SignalP-5.0 (https://services.healthtech.dtu.dk/services/SignalP-5.0/, accessed on 3 September 2025) for signal peptide and cleavage site prediction; and RNAfold (http://rna.tbi.univie.ac.at/cgi-bin/RNAWebSuite/RNAfold.cgi, accessed on 3 September 2025) for RNA structure prediction.

## 4. Conclusions

Current progress in adoptive cell therapy has demonstrated the validity of the technology which has continued to advance and improve. Despite its general limitation to the treatment of blood cancers at present, tremendous efforts are underway to investigate its application in other incurable diseases, including multiple sclerosis, arthritis, and lupus. For some of these indications, mRNA-CAR-T has emerged as a potentially safer and perhaps cheaper option. Currently, there are more than 10 mRNA-based CAR therapies undergoing clinical evaluation [84]. The same group that developed the Comirnaty™ vaccine has used an mRNA vaccine to boost CAR-T cell efficacy in solid tumours [85]. IVT mRNAs are currently being used in trials by several biotech companies [86,87,88]. In general, these mRNAs are chemically modified to improve transcript stability.

In this study, we explored the significance of m6A sites in both the coding region and UTRs of a transiently transfected IVT CD19-CAR in T cells. Notably, the presence of DRACH consensus sites in the coding region of the transcript acted as substrates for methyltransferase activity upon entry into the cytoplasm. Eliminating those sites resulted in lower and less-stable expression of the CAR protein. Adding m6A consensus sites in the UTRs had, in some cases, additional benefits to protein yield; however, this was not always in an m6A-dependent manner. As such, the methylation of consensus m6A sites seems to be sequence context-dependent. It remains to be determined to what extent engineering further DRACH sites into a particular transcript, or into what region of the transcript, will benefit stability or translation as the complexities of secondary structure and the cohort of reader proteins expressed in a particular cell will likely dictate the outcome. Further studies to establish the relationship between secondary structure and which m6A readers are responsible for mediating a particular fate will aid in future epitranscriptomic engineering strategies.

## Figures and Tables

**Table 1:** Impact of various 5′ and 3′ UTRs on CD19-CAR expression. Predicted m6A sites are red; Median Fluorescence Intensity (MFI); Minimum Free Energy (MFE).

Vectors	Sequence (5’-3’); Number of Predicted and Confidence Score of m6A Sites and Secondary Features			Normalised MFI to min-CAR	
**5’ UTR**		**Number of predicted m6A (SRAMP score)**	MFE	24 h	48 h
**P-CAR**	GAGAATAAACTAGTATTCTTCTGGTCCCCACAGACTCAGAGAGAACCC	1 (moderate)	−10.66	1.03	1.01
**O-CAR**	AAAAAAAAAAAACCAAAAAAAAAAAACAAAAAAAAAAAATAATTGACTAA		−0.08	0.81	0.73
**2M6-CAR**	GGACTAAAGCGGACTTGT	2 (both high score)	−2.6	1.16	1.21
**F-CAR**	CTGAAACACGGTGGAGAGTTTATTGCAAAATAACGCGTCCATTCGACA		−9.4	1.15	1.22
**APT-CAR**	ACTCACTATTTGTTTTCGCGCCCAGTTGCAAAAAGTGTCG		−6.15	1.07	1.11
**Positive Control**					
**PFT-CAR**	This includes 5’ UTR of P-CAR and P-3’ UTR as shown below TCTGGTACTGCATGCACGCAATGCTAGCTGCCCCTTTCCCGTCCTGGGTACCCCGAGTCTCCCCCGACCTCGGGTCCCAGGTATGCTCCCACCTCCACCTGCCCCACTCACCACCTCTGCTAGTTCCAGACACCTCCCAAGCACGCAGCAATGCAGCTCAAAACGCTTAGCCTAGCCACACCCCCACGGGAAACAGCAGTGATTAACCTTTAGCAATAAACGAAAGTTTAACTAAGCTATACTAACCCCAGGGTTGGTCAATTTCGTGCCAGCCAC	1 (moderate) in 5’ UTR		1.17	1.24
**3’ UTR**					
**CAR-PARA**	TGATATATTTTAACACCCTAGGTTAATTAACTAGTATAAAA		−3.06	1.19	1.16
**CAR-M6**	T GGACT TATATTTTAACACCCTAGGTTAATTAACTAGTAT	1 (very high score)	−3.71	1.17	1.15

**Table 2:** Summary of the impact of different 3′ UTRs on CD19-CAR expression and predicted m6A methylation sites. Predicted m6A sites are in red, vsg 16-mer are in green; Median Fluorescence Intensity (MFI); Minimum Free Energy (MFE); duplicate SV40 elements are underlined; AT-rich motif bound by the human antigen R (HuR) protein is in amber; predicted target sites (score from 97 to 100) for human miR-4719 are in bold; m6A qPCR: methylation-sensitive quantitative RT-PCR.

Vectors	3’ UTR Sequences (5’-3’)	Number of Predicted m6A Sites (SRAMP Score)	MFE	Normalised MFI to min-CAR	M6A qPCR
					
CAR-2M6-2v16	GGACTAAAGCGGACTGAAAATGATATATTTTAACACCTCCTGATATATTTTAACACAAAA	2 (1st site: very high score; 2nd site: high score)	−3.66	1.403	No methylation
CAR-2M6L-2v16	GGACTAAAGCGGACTTGTGCCACCTGATATATTTTAACACGAAAACTCCTGATATATTTTAACACAAAA	2 (1st site: very high score; 2nd site: high score)	−6.79	1.372	No methylation
CAR-W3SL	TCAACCTCTGGATTACAAA**ATTTGTGA**AAGATTGACTGGTATTCTTAACTATGTTGCTCCTTTTACGCTATGTGGATACGCTGCTTTAATGCCTTTGTATCATGCTATTGCTTCCCGTATGGCTTTCATTTTCTCCTCCTTGTATAAATCCTGGTTAGTTCTTGCCACGGCGGAACTCATCGCCGCCTGCCTTGCCCGCTGCTGGACAGGGGCTCGGCTGTTGGGCACTGACAATTCCGTGGTGTTT**ATTTGTGA**A**ATTTGTGA**TGCTATTGCTTTATTTGTAACCATCTAGCTTT**ATTTGTGA**A**ATTTGTGA**TGCTATTGCTTTATTTGTAACCATTATAAGCTGCAATAAACAAGTTAACAACAACAATTGCATTCATTTTATGTTTCAGGTTCAGGGGGAGATGTGGGAGGTTTTTTAAAGCG		−114	1.018	
CAR-W3SL2M6v16	TCAACCTCTGGATTACAAA**ATTTGTGA**AAGATTGACTGGTATTCTTAACTATGTTGCTCCTTTTACGCTATGTGGATACGCTGCTTTAATGCCTTTGTATCATGCTATTGCTTCCCGTATGGCTTTCATTTTCTCCTCCTTGTATAAATCCTGGTTAGTTCTTGCCACGGCGGAACTCATCGCCGCCTGCCTTGCCCGCTGCTGGACAGGGGCTCGGCTGTTGGGCACTGACAATTCCGTGGTGTTT**ATTTGTGA**A**ATTTGTGA**TGCTATTGCTTTATTTGTAACCATCTAGCTTT**ATTTGTGA**A**ATTTGTGA**TGCTATTGCTTTATTTGTAACCATTATAAGCTGCAATAAACAAGTTAACAACAACAATTGCATTCATTTTATGTTTCAGGTTCAGGGGGAGATGTGGGAGGTTTTTTAAAGCGTAAGGACTAAAGCGGACTGAAAACTCCTGATATATTTTAACACAAAA	2 (both are very high score)	−119	1.023	No methylation
CAR-2M6v16SL	GGACTAAAGCGGACTGAAAACTCCTGATATATTTTAACACT**ATTTGTGA**A**ATTTGTGA**TGCTATTGCTTTATTTGTAACCATCTAGCTTT**ATTTGTGA**A**ATTTGTGA**TGCTATTGCTTTATTTGTAACCATTATAAGCTGCAATAAACAAGTTAACAACAACAATTGCATTCATTTTATGTTTCAGGTTCAGGGGGAGATGTGGGAGGTTTTTTAAAGCG	2 (1st site: very high score; 2nd site: high score)	−41.7	0.72	No methylation
2M6-CAR-2M6-v16-SL70	GGACTAAAGCGGACTGAAAACTCCTGATATATTTTAACACT**ATTTGTGA**A**ATTTGTGA**TGCTATTGCTTTATTTGTAACCATCTAGCTTT**ATTTGTGA**A**ATTTGTGA**TGCTATTGCTTTATTTGTAACCATTATAAGCTGCAATAAACAAGTTAACAACAACAATTGCATTCATTTTATGTTTCAGGTTCAGGGGGAGATGTGGGAGGTTTTTTAAAGCGAAAATTTA	2 at the 5’ UTR (high score); 2 at the 3’ UTR (1st site: very high score; 2nd site: high score	−41.7	0.884	Not measured

**Table 3:** Summary of impact of various vsg 3′ UTR-derived constructs on CD19-CAR expressions. Predicted m6A sites are red; vsg-16-mers are green; vsg-8-mers are blue. Median Fluorescence Intensity (MFI); Minimum Free Energy (MFE); m6A quantitative RT-PCR (m6A-qPCR).

Vectors	Sequences (5’-3’); Number of Predicted and Confidence Score of m6A Sites and Secondary Features			Normalised MFI to min-CAR	M6A qPCR
	**Sequence Between Stop of CAR (TAAA) and polyA**	**Number of Predicted m6A Sites (SRAMP Score)**	**MFE**		
**CAR-vsg3UTR**	TTTTCCCCCTCTTTTTCTTAAAAATTCTTGCTACTTGAAAACTCCTGATATATTTTAACAC		−1.16	1.28	not measured
**CAR-2M6vsg3UTR**	GGACTAAAGCGGACTTGTGCCACCTTTTCCCCCTCTTTTTCTTAAAAATTCTTGCTACTTGAAAACTCCTGATATATTTTAACAC	2 (high score)	−5.02	1.21	Methylation at both sites
**CAR-8R2M6vsg3UTR**	TTTTCCCCCTCTTTTTCTTAAAAATTCGGACTAAAGCGGACTGAAAACTCCTGATATATTTTAACAC	2 (high score)	−6.07	1.43	no methylation

## Data Availability

Data are available from the corresponding authors upon reasonable request.

## Supplementary Materials

The following supporting information can be downloaded at: https://www.mdpi.com/article/10.3390/ijms27020796/s1.

## References

[1] Żak M.M., Zangi L. (2025). Clinical development of therapeutic mRNA applications. Mol. Ther..

[2] Xiao Y., Wu B., Zhou Q., Wu F., Li N., Xu C., Han X. (2025). Technologies for chimeric antigen receptor transgene delivery. Trends Mol. Med..

[3] Emily Whitehead: A Young Girl Beats Cancer with Immunotherapy. https://www.cancerresearch.org/stories/patients/emily-whitehead.

[4] Wang M., Jia L., Dai X., Zhang X. (2024). Advanced strategies in improving the immunotherapeutic effect of CAR-T cell therapy. Mol. Oncol..

[5] Willyard C. (2024). Do cutting-edge CAR-T-cell therapies cause cancer? What the data say. Nature.

[6] Guffroy A., Jacquel L., Guffroy B., Martin T. (2024). CAR-T cells for treating systemic lupus erythematosus: A promising emerging therapy. Joint Bone Spine.

[7] Harrison C. (2024). CAR-Ts sweep into autoimmunity. Nat. Biotechnol..

[8] Richter J., Fischbach F., Pfeffer L.K., Fehse B., Berger S.C., Reinhardt S., Schäfersküpper M., Marquard F.E., Rathje K., Gagelmann N. (2024). CD19-Directed CAR T Cell Therapy in 4 Patients with Refractory Multiple Sclerosis. Blood.

[9] Pieper T., Roth K.D.R., Glaser V., Riet T., Buitrago-Molina L.E., Hagedorn M., Lieber M., Hust M., Noyan F., Jaeckel E. (2023). Generation of Chimeric Antigen Receptors against Tetraspanin 7. Cells.

[10] Beatty G.L., Haas A.R., Maus M.V., Torigian D.A., Soulen M.C., Plesa G., Chew A., Zhao Y., Levine B.L., Albelda S.M. (2014). Mesothelin-specific chimeric antigen receptor mRNA-engineered T cells induce anti-tumor activity in solid malignancies. Cancer Immunol. Res..

[11] Wu D., Xu-Monette Z.Y., Zhou J., Yang K., Wang X., Fan Y., Young K.H. (2025). CAR T-cell therapy in autoimmune diseases: A promising frontier on the horizon. Front. Immunol..

[12] Bonini C., Chapuis A.G., Hudecek M., Guedan S., Magnani C.F., Qasim W. (2023). Genome Editing in Engineered T Cells for Cancer Immunotherapy. Hum. Gene Ther..

[13] Cappabianca D., Li J., Zheng Y., Tran C., Kasparek K., Mendez P., Thu R., Maures T., Capitini C.M., Deans R. (2024). Non-viral expression of chimeric antigen receptors with multiplex gene editing in primary T cells. Front. Bioeng. Biotechnol..

[14] Gehrke L., Gonçalves V.D.R., Andrae D., Rasko T., Ho P., Einsele H., Hudecek M., Friedel S.R. (2024). Current Non-Viral-Based Strategies to Manufacture CAR-T Cells. Int. J. Mol. Sci..

[15] Sahin U., Karikó K., Türeci Ö. (2014). mRNA-based therapeutics—Developing a new class of drugs. Nat. Rev. Drug Discov..

[16] Rohner E., Yang R., Foo K.S., Goedel A., Chien K.R. (2022). Unlocking the promise of mRNA therapeutics. Nat. Biotechnol..

[17] Henderson J.M., Ujita A., Hill E., Yousif-Rosales S., Smith C., Ko N., McReynolds T., Cabral C.R., Escamilla-Powers J.R., Houston M.E. (2021). Cap 1 messenger RNA synthesis with co-transcriptional cleancap^®^ analog by in-vitro transcription. Curr. Protoc..

[18] Chen H., Liu D., Guo J., Aditham A., Zhou Y., Tian J., Luo S., Ren J., Hsu A., Huang J. (2025). Branched chemically modified poly (A) tails enhance the translation capacity of mRNA. Nat. Biotechnol..

[19] Xia X. (2021). Detailed dissection and critical evaluation of the Pfizer/BioNTech and Moderna mRNA vaccines. Vaccines.

[20] Lewis C.J.T., Xie L.H., Bhandarkar S.M., Jin D., Abdallah K., Draycott A.S., Chen Y., Thoreen C.C., Gilbert W.V. (2025). Quantitative profiling of human translation initiation reveals elements that potently regulate endogenous and therapeutically modified mRNAs. Mol. Cell.

[21] Trepotec Z., Aneja M.K., Geiger J., Hasenpusch G., Plank C., Rudolph C. (2019). Maximizing the translational yield of mRNA therapeutics by minimizing 5′-UTRs. Tissue Eng. Part A.

[22] Li T., Liu G., Bu G., Xu Y., He C., Zhao G. (2025). Optimizing mRNA translation efficiency through rational 5′ UTR and 3′ UTR combinatorial design. Gene.

[23] Seo J.J., Jung S.J., Yang J., Choi D.E., Kim V.N. (2023). Functional viromic screens uncover regulatory RNA elements. Cell.

[24] Jung S.-J., Seo J.J., Lee S., Hyun S.I., Lee J.E., Lee S., Lee Y., Chang H., Lee H., Kim J.H. (2025). RNA stability enhancers for durable base-modified mRNA therapeutics. Nat. Biotechnol..

[25] Dousis A., Ravichandran K., Hobert E.M., Moore M.J., Rabideau A.E. (2023). An engineered T7 RNA polymerase that produces mRNA free of immunostimulatory byproducts. Nat. Biotechnol..

[26] Chen H., Liu D., Aditham A., Guo J., Huang J., Kostas F., Maher K., Friedrich M.J., Xavier R.J., Zhang F. (2025). Chemical and topological design of multicapped mRNA and capped circular RNA to augment translation. Nat. Biotechnol..

[27] Lu G., Shivalila C., Monian P., Yu H., Harding I., Briem S., Byrne M., Faraone A., Friend S., Huth O. (2024). Rational design of base, sugar and backbone modifications improves ADAR-mediated RNA editing. Nucleic Acids Res..

[28] Dominissini D., Moshitch-Moshkovitz S., Schwartz S., Salmon-Divon M., Ungar L., Osenberg S., Cesarkas K., Jacob-Hirsch J., Amariglio N., Kupiec M. (2012). Topology of the human and mouse m6A RNA methylomes revealed by m6A-seq. Nature.

[29] Meyer K.D., Saletore Y., Zumbo P., Elemento O., Mason C.E., Jaffrey S.R. (2012). Comprehensive analysis of mRNA methylation reveals enrichment in 3′ UTRs and near stop codons. Cell.

[30] Clinical Trials: Testing the Safety and Efficacy of STC-15, First-in-Class METTL3 Inhibitor. https://www.stormtherapeutics.com/clinical-trials/.

[31] Du H., Zhao Y., He J., Zhang Y., Xi H., Liu M., Ma J., Wu L. (2016). YTHDF2 destabilizes m6A-containing RNA through direct recruitment of the CCR4–NOT deadenylase complex. Nat. Commun..

[32] Zhou Y., Ćorović M., Hoch-Kraft P., Meiser N., Mesitov M., Körtel N., Back H., Naarmann-de Vries I.S., Katti K., Obrdlík A. (2024). m6A sites in the coding region trigger translation-dependent mRNA decay. Mol. Cell.

[33] Lemm I., Ross J. (2022). Regulation of c-myc mRNA decay by translational pausing in a coding region instability determinant. Mol. Cell. Biol..

[34] Ćorović M., Hoch-Kraft P., Zhou Y., Hallstein S., König J., Zarnack K. (2025). m6A in the coding sequence: Linking deposition, translation, and decay. Trends Genet..

[35] Linder B., Sharma P., Wu J., Birbaumer T., Eggers C., Murakami S., Ott R.E., Fenzl K., Vorgerd H., Erhard F. (2025). tRNA modifications tune m6A-dependent mRNA decay. Cell.

[36] Murakami S., Olarerin-George A.O., Liu J.F., Zaccara S., Hawley B., Jaffrey S.R. (2025). m6A alters ribosome dynamics to initiate mRNA degradation. Cell.

[37] Costello A., Lao N.T., Barron N., Clynes M. (2019). Improved yield of rhEPO in CHO cells with synthetic 5′ UTR. Biotechnol. Lett..

[38] Costello A., Lao N.T., Barron N., Clynes M. (2020). Reinventing the wheel: Synthetic circular RNAs for mammalian cell engineering. Trends Biotechnol..

[39] Lao N., Barron N. (2023). Enhancing recombinant protein and viral vector production in mammalian cells by targeting the YTHDF readers of N6-methyladenosine in mRNA. Biotechnol. J..

[40] Kavanagh H., Dunne S., Martin D.S., McFadden E., Gallagher L., Schwaber J., Leonard S., O'Dea S. (2021). A novel non-viral delivery method that enables efficient engineering of primary human T cells for ex vivo cell therapy applications. Cytotherapy.

[41] Wang Z., Li P., Zeng X., Guo J., Zhang C., Fan Z., Wang Z., Zhu P., Chen Z. (2025). CAR-T therapy dilemma and innovative design strategies for next generation. Cell Death Dis..

[42] Zhou Y., Zeng P., Li Y.H., Zhang Z., Cui Q. (2016). SRAMP: Prediction of mammalian N6-methyladenosine (m6A) sites based on sequence-derived features. Nucleic Acids Res..

[43] Yankova E., Blackaby W., Albertella M., Rak J., De Braekeleer E., Tsagkogeorga G., Pilka E.S., Aspris D., Leggate D., Hendrick A.G. (2021). Small-molecule inhibition of METTL3 as a strategy against myeloid leukaemia. Nature.

[44] Xiao H., Zhao R., Meng W., Liao Y. (2023). Effects and translatomics characteristics of a small-molecule inhibitor of METTL3 against non-small cell lung cancer. J. Pharm. Anal..

[45] Schöller E.V.A., Weichmann F., Treiber T., Ringle S., Treiber N., Flatley A., Feederle R., Bruckmann A., Meister G. (2018). Interactions, localization, and phosphorylation of the m6A generating METTL3–METTL14–WTAP complex. RNA.

[46] Lin S., Choe J., Du P., Triboulet R., Gregory R.I. (2016). The m6A methyltransferase METTL3 promotes translation in human cancer cells. Mol. Cell.

[47] Gokhale N.S., McIntyre A.B., McFadden M.J., Roder A.E., Kennedy E.M., Gandara J.A., Hopcraft S.E., Quicke K.M., Vazquez C., Willer J. (2016). N6-methyladenosine in flaviviridae viral RNA genomes regulates infection. Cell Host Microbe.

[48] Sacco M.T., Bland K.M., Horner S.M. (2022). WTAP Targets the METTL3 m^6^A-Methyltransferase Complex to Cytoplasmic Hepatitis C Virus RNA to Regulate Infection. J. Virol..

[49] Sattar S., Kabat J., Jerome K., Feldmann F., Bailey K., Mehedi M. (2023). Nuclear translocation of spike mRNA and protein is a novel feature of SARS-CoV-2. Front. Microbiol..

[50] De La Cruz B.M., Darsinou M., Riccio A. (2023). From form to function: M6A methylation links mRNA structure to metabolism. Adv. Biol. Regul..

[51] Mao Y., Dong L., Liu X.M., Guo J., Ma H., Shen B., Qian S.B. (2019). m^6^A in mRNA coding regions promotes translation via the RNA helicase-containing YTHDC2. Nat. Commun..

[52] Kumar A., Daripa P., Penumutchu S., Maiti S., Jain N. (2025). Thermodynamic insights into N6-methyladenosine-modified ribonucleic acids and their interactions with the RNA recognition motif of heterogeneous nuclear ribonucleoprotein C. Int. J. Biol. Macromol..

[53] Zaccara S., Jaffrey S.R. (2024). Understanding the redundant functions of the m6A-binding YTHDF proteins. RNA.

[54] Xu L., Yu Q., Peihang X., Li K., Wang B., Shao Y., Cheng M., Huang W., Yao Q., Feng X. (2025). YTHDF3 promotes breast cancer osteolytic bone metastasis by enhancing the translation of ZEB1 and SMAD5. Oncogenesis.

[55] Hao L., Wang J.M., Liu B.Q., Yan J., Li C., Jiang J.Y., Zhao F.Y., Qiao H.Y., Wang H.Q. (2021). m6A-YTHDF1-mediated TRIM29 upregulation facilitates the stem cell-like phenotype of cisplatin-resistant ovarian cancer cells. Biochim. Biophys. Acta (BBA)-Mol. Cell Res..

[56] Chang G., Shi L., Ye Y., Shi H., Zeng L., Tiwary S., Huse J.T., Huo L., Ma L., Ma Y. (2020). YTHDF3 induces the translation of m6A-enriched gene transcripts to promote breast cancer brain metastasis. Cancer Cell.

[57] Huang H., Weng H., Sun W., Qin X., Shi H., Wu H., Zhao B.S., Mesquita A., Liu C., Yuan C.L. (2018). Recognition of RNA N^6^-methyladenosine by IGF2BP proteins enhances mRNA stability and translation. Nat. Cell Biol..

[58] Coyne S., Wu T., Hossain M., Vinjamur D., Starrs M., Zeng J., Andresen F., Gutierrez A., Shimamura A., Bauer D.E. (2025). Direct and indirect regulation of fetal globin transcript by RNA-binding protein IGF2BP1. bioRxiv.

[59] Liu S., Liao S., He J., Zhou Y., He Q. (2025). IGF2BP2: An m6A reader that affects cellular function and disease progression. Cell. Mol. Biol. Lett..

[60] Xiong X., Wang X., Wang Z., Chen F., Shi Y., Chen X., Gong W., Jia X., Xu J. (2025). IGF2BP2 promotes malignant progression of ovarian cancer by regulating protein synthesis through liquid-liquid phase separation. Cell Rep..

[61] Zhou C., Wang M., Du X., Xue L., Zhu X., Li X., Zhao Q. (2025). WTAP/IGF2BP3 Mediated m6A Modification of SOD2 mRNA Aggravates the Tumourigenesis of Colorectal Cancer. J. Biochem. Mol. Toxicol..

[62] Kath J., Du W., Pruene A., Braun T., Thommandru B., Turk R., Sturgeon M.L., Kurgan G.L., Amini L., Stein M. (2022). Pharmacological interventions enhance virus-free generation of TRAC-replaced CAR T cells. Mol. Ther. Methods Clin. Dev..

[63] Fan R., Cui C., Kang B., Chang Z., Wang G., Cui Q. (2024). A combined deep learning framework for mammalian m6A site prediction. Cell Genom..

[64] Garalde D.R., Snell E.A., Jachimowicz D., Sipos B., Lloyd J.H., Bruce M., Pantic N., Admassu T., James P., Warland A. (2018). Highly parallel direct RNA sequencing on an array of nanopores. Nat. Methods.

[65] Castellanos-Rubio A., Santin I., Olazagoitia-Garmendia A., Romero-Garmendia I., Jauregi-Miguel A., Legarda M., Bilbao J.R. (2019). A novel RT-QPCR-based assay for the relative quantification of residue specific m6A RNA methylation. Sci. Rep..

[66] Meyer K.D., Patil D.P., Zhou J., Zinoviev A., Skabkin M.A., Elemento O., Pestova T.V., Qian S.B., Jaffrey S.R. (2015). 5′ UTR m6A promotes cap-independent translation. Cell.

[67] Park O.H., Ha H., Lee Y., Boo S.H., Kwon D.H., Song H.K., Kim Y.K. (2019). Endoribonucleolytic cleavage of m6A-containing RNAs by RNase P/MRP complex. Mol. Cell.

[68] Shi H., Wang X., Lu Z., Zhao B.S., Ma H., Hsu P.J., Liu C., He C. (2017). YTHDF3 facilitates translation and decay of N6-methyladenosine-modified RNA. Cell Res..

[69] Zou Z., He C. (2024). The YTHDF proteins display distinct cellular functions on m^6^A-modified RNA. Trends Biochem. Sci..

[70] Chen R., Wang S.K., Belk J.A., Amaya L., Li Z., Cardenas A., Abe B.T., Chen C.K., Wender P.A., Chang H.Y. (2023). Engineering circular RNA for enhanced protein production. Nat. Biotechnol..

[71] Tusup M., French L.E., Guenova E., Kundig T.M., Pascolo S. (2018). Optimizing the Functionality of in vitro-Transcribed mRNA. Biomed. J. Sci. Tech. Res..

[72] Sample P.J., Wang B., Reid D.W., Presnyak V., McFadyen I.J., Morris D.R., Seelig G. (2019). Human 5′ UTR design and variant effect prediction from a massively parallel translation assay. Nat. Biotechnol..

[73] Linares-Fernández S., Moreno J., Lambert E., Mercier-Gouy P., Vachez L., Verrier B., Exposito J.Y. (2021). Combining an optimized mRNA template with a double purification process allows strong expression of in vitro transcribed mRNA. Mol. Ther. Nucleic Acids.

[74] Wang X., Zhao B.S., Roundtree I.A., Lu Z., Han D., Ma H., Weng X., Chen K., Shi H., He C. (2015). N6-methyladenosine modulates messenger RNA translation efficiency. Cell.

[75] Viegas I.J., de Macedo J.P., Serra L., De Niz M., Temporão A., Silva Pereira S., Mirza A.H., Bergstrom E., Rodrigues J.A., Aresta-Branco F. (2022). N 6-methyladenosine in poly (A) tails stabilize VSG transcripts. Nature.

[76] von Niessen A.G.O., Poleganov M.A., Rechner C., Plaschke A., Kranz L.M., Fesser S., Diken M., Löwer M., Vallazza B., Beissert T. (2019). Improving mRNA-based therapeutic gene delivery by expression-augmenting 3′ UTRs identified by cellular library screening. Mol. Ther..

[77] Choi J.-H., Yu N.K., Baek G.C., Bakes J., Seo D., Nam H.J., Baek S.H., Lim C.S., Lee Y.S., Kaang B.K. (2014). Optimization of AAV expression cassettes to improve packaging capacity and transgene expression in neurons. Mol. Brain.

[78] Schambach A., Galla M., Maetzig T., Loew R., Baum C. (2007). Improving transcriptional termination of self-inactivating gamma-retroviral and lentiviral vectors. Mol. Ther..

[79] Ma X., Liu S., Fan B., Jin D., Miao L., Liu L., Du S., Lin J. (2025). Enhancing mRNA translation efficiency by introducing sequence optimized AU rich elements in 3’UTR via HuR anchorage. Mol. Ther. Nucleic Acids.

[80] Behm-Ansmant I., Rehwinkel J., Doerks T., Stark A., Bork P., Izaurralde E. (2006). mRNA degradation by miRNAs and GW182 requires both CCR4: NOT deadenylase and DCP1: DCP2 decapping complexes. Genes Dev..

[81] Ridewood S., Ooi C.P., Hall B., Trenaman A., Wand N.V., Sioutas G., Scherwitzl I., Rudenko G. (2017). The role of genomic location and flanking 3′ UTR in the generation of functional levels of variant surface glycoprotein in Trypanosoma brucei. Mol. Microbiol..

[82] Melo do Nascimento L., Egler F., Arnold K., Papavasiliou N., Clayton C., Erben E. (2021). Functional insights from a surface antigen mRNA-bound proteome. eLife.

[83] Madeira F., Madhusoodanan N., Lee J., Eusebi A., Niewielska A., Tivey A.R., Lopez R., Butcher S. (2024). The EMBL-EBI Job Dispatcher sequence analysis tools framework in 2024. Nucleic Acids Res..

[84] Wu J., Wu W., Zhou B., Li B. (2024). Chimeric antigen receptor therapy meets mRNA technology. Trends Biotechnol..

[85] Mackensen A., Haanen J.B., Koenecke C., Alsdorf W., Wagner-Drouet E., Borchmann P., Heudobler D., Ferstl B., Klobuch S., Bokemeyer C. (2023). CLDN6-specific CAR-T cells plus amplifying RNA vaccine in relapsed or refractory solid tumors: The phase 1 BNT211-01 trial. Nat. Med..

[86] Carvalho T. (2023). Personalized anti-cancer vaccine combining mRNA and immunotherapy tested in melanoma trial. Nat. Med..

[87] Miljkovic M.D., Asch A.S., Orloff G., Boccia R., Berdeja J.G., Altuntas F., Ciurea S.O., Howard J.F., Vu T., Myers B. (2024). Safety and Tolerability of BCMA-Directed mRNA CAR T-Cell Therapy in Multiple Myeloma and Autoimmune Disease. Blood.

[88] Sun Y., Chatterjee S., Lian X., Traylor Z., Sattiraju S.R., Xiao Y., Dilliard S.A., Sung Y.C., Kim M., Lee S.M. (2024). In vivo editing of lung stem cells for durable gene correction in mice. Science.

