# Supplementary material for: m6A-Modified Nucleotide Bases Improve Translation of In Vitro-Transcribed Chimeric Antigen Receptor (CAR) mRNA in T Cells
Source: Int J Mol Sci. 2026 Jan 13;27(2):796. doi: 10.3390/ijms27020796 (PMC12841529; doi:10.3390/ijms27020796)
Supplement: Supplementary file 1 [file ijms-27-00796-s001.zip › Suppl_data.pdf]

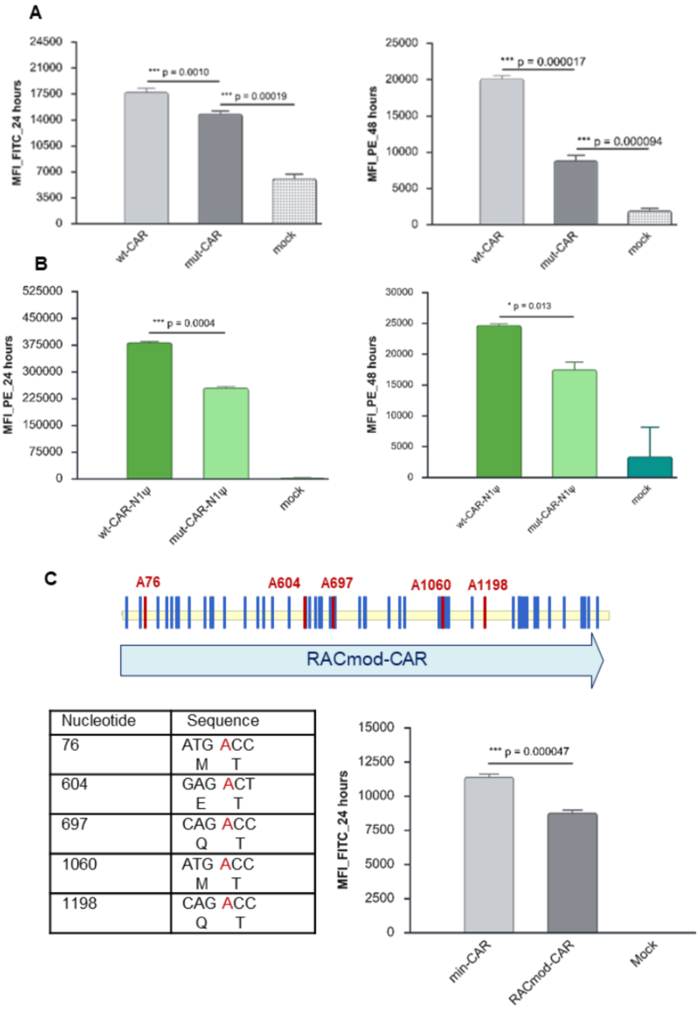

Nucleotide	Sequence
76	ATG ACC<br>M T
604	GAG ACT<br>E T
697	CAG ACC<br>Q T
1060	ATG ACC<br>M T
1198	CAG ACC<br>Q T

Figure S1: Modification of predicted m6A sites negatively impacts CD19-CAR expression. (A) Relative Median Fluorescence Intensity (MFI) of activated T cells transiently transfected with CD19-CAR mRNAs synthesized with uridine after 24 hours (left panel) and 48 hours (right panel) (n = 3 biological replicates; n = 3 technical replicates); (B) MFI of activated T cells transiently transfected with CD19-CAR mRNAs synthesized with N1-methyl-pseudouridine (N1Ψ) (n = 2 biological replicates; n = 2 technical replicates); (+/-SD); * (p<0.05); ** (p<0.01), and *** (p<0.001) (two-sided Student's *t*-test. (C) CD19-CAR transcript and synonymous changes of RAC sites represented by vertical blue lines. Five non-modified RAC sequences shown as red vertical lines. List of nucleotide and amino acid sequences at five non-modified RAC in RACmod-CAR sequence (left); Relative Median Fluorescence Intensity (MFI) of activated T cells transiently transfected with min-CAR and RACmod-CAR mRNAs 24 hours post electroporation (n = 2 biological replicates; n = 3 technical replicates) (right); (+/-SD); * indicates p<0.05; ** indicates p<0.01, and *** indicates p<0.001 (Two-sided Student's *t*-test). Graphs and figure created at https://BioRender.com.

```
TRAC-CAR MALPVTAL-LLPLALLLHAARPDIQMTQTTSSLSASLGDRVTISCRASQDISKYLNWYQQ 59
Wt-CAR   MALPVTAL-LLPLALLLHAARPDIQMTQTTSSLSASLGDRVTISCRASQDISKYLNWYQQ 59
HM852952 MLLLVTSLLLCELPHPAFLLIPDIQMTQTTSSLSASLGDRVTISCRASQDISKYLNWYQQ 60
         * * **:* *  *    .   ***************************************

TRAC-CAR KPDGTVKLLIYHTSRLHSGVPSRFSGSGSGTDYSLTISNLEQEDIATYFCQQGNTLPYTF 119
Wt-CAR   KPDGTVKLLIYHTSRLHSGVPSRFSGSGSGTDYSLTISNLEQEDIATYFCQQGNTLPYTF 119
HM852952 KPDGTVKLLIYHTSRLHSGVPSRFSGSGSGTDYSLTISNLEQEDIATYFCQQGNTLPYTF 120
         ************************************************************

TRAC-CAR GGGTKLEITGGG---GSGGGGSGGGGSEVKLQESGPGLVAPSQSLSVTCTVSGVSLPDYG 176
Wt-CAR   GGGTKLEITGSTSGSGKPGSGEGSTKGEVKLQESGPGLVAPSQSLSVTCTVSGVSLPDYG 179
HM852952 GGGTKLEITGSTSGSGKPGSGEGSTKGEVKLQESGPGLVAPSQSLSVTCTVSGVSLPDYG 180
         **********.    *. *.*.*.  .*********************************

TRAC-CAR VSWIRQPPRKGLEWLGVIWGSETTYYNSALKSRLTIIKDNSKSQVFLKMNSLQTDDTAIY 236
Wt-CAR   VSWIRQPPRKGLEWLGVIWGSETTYYNSALKSRLTIIKDNSKSQVFLKMNSLQTDDTAIY 239
HM852952 VSWIRQPPRKGLEWLGVIWGSETTYYNSALKSRLTIIKDNSKSQVFLKMNSLQTDDTAIY 240
         ************************************************************

TRAC-CAR YCAKHYYYGGSYAMDYWGQGTSVTVSS 267
Wt-CAR   YCAKHYYYGGSYAMDYWGQGTSVTVSS 266
HM852952 YCAKHYYYGGSYAMDYWGQGTSVTVSS 263
         ***************************

TRAC-CAR                               FWVLVVVGGVLACYSLLVTVAFIIFWVRSK 416
Wt-CAR                                 FWVLVVVGGVLACYSLLVTVAFIIFWVRSK 341
HM852952                               FWVLVVVGGVLACYSLLVTVAFIIFWVRSK 339
                                       ******************************
                                                      4-1BB
TRAC-CAR RSRLLHSDYMNMTPRRPGPTRKHYQPYAPPRDFAAYRS---------------------- 454
Wt-CAR   RSRLLHSDYMNMTPRRPGPTRKHYQPYAPPRDFAAYRSKRGRKKLLYIFKQPFMRPVQTT 401
HM852952 RSRLLHSDYMNMTPRRPGPTRKHYQPYAPPRDFAAYRS---------------------- 377
         **************************************
                                          *
TRAC-CAR --------------------RVKFSRSADAPAYQQGQNQLYNELNLGRREEYDVLDKRRG 494
Wt-CAR   QEEDGCSCRFPEEEEGGCELRVKFSRSADAPAYKQGQNQLYNELNLGRREEYDVLDKRRG 461
HM852952 --------------------RVKFSRSADAPAYQQGQNQLYNELNLGRREEYDVLDKRRG 417
                             *************:**************************

TRAC-CAR RDPEMGGKPRRKNPQEGLYNELQKDKMAEAYSEIGMKGERRRGKGHDGLYQGLSTATKDT 554
Wt-CAR   RDPEMGGKPRRKNPQEGLYNELQKDKMAEAYSEIGMKGERRRGKGHDGLYQGLSTATKDT 521
HM852952 RDPEMGGKPRRKNPQEGLYNELQKDKMAEAYSEIGMKGERRRGKGHDGLYQGLSTATKDT 477
         ************************************************************

TRAC-CAR YDALHMQALPPR 566
Wt-CAR   YDALHMQALPPR 533
HM852952 YDALHMQALPPR 489
         ************
```

Figure S2. Multiple alignment of CD19-CAR protein sequence. Multiple alignment of Antigen recognition domain (top); of CD28 transmembrane and intracellular domain and CD3-ζ domains (bottom). Cleavage site between amino acid 21-22 is shown as blue arrowhead; Signal peptide: red letters; linker: blue letters; different amino acid: red star above the alignment.

A

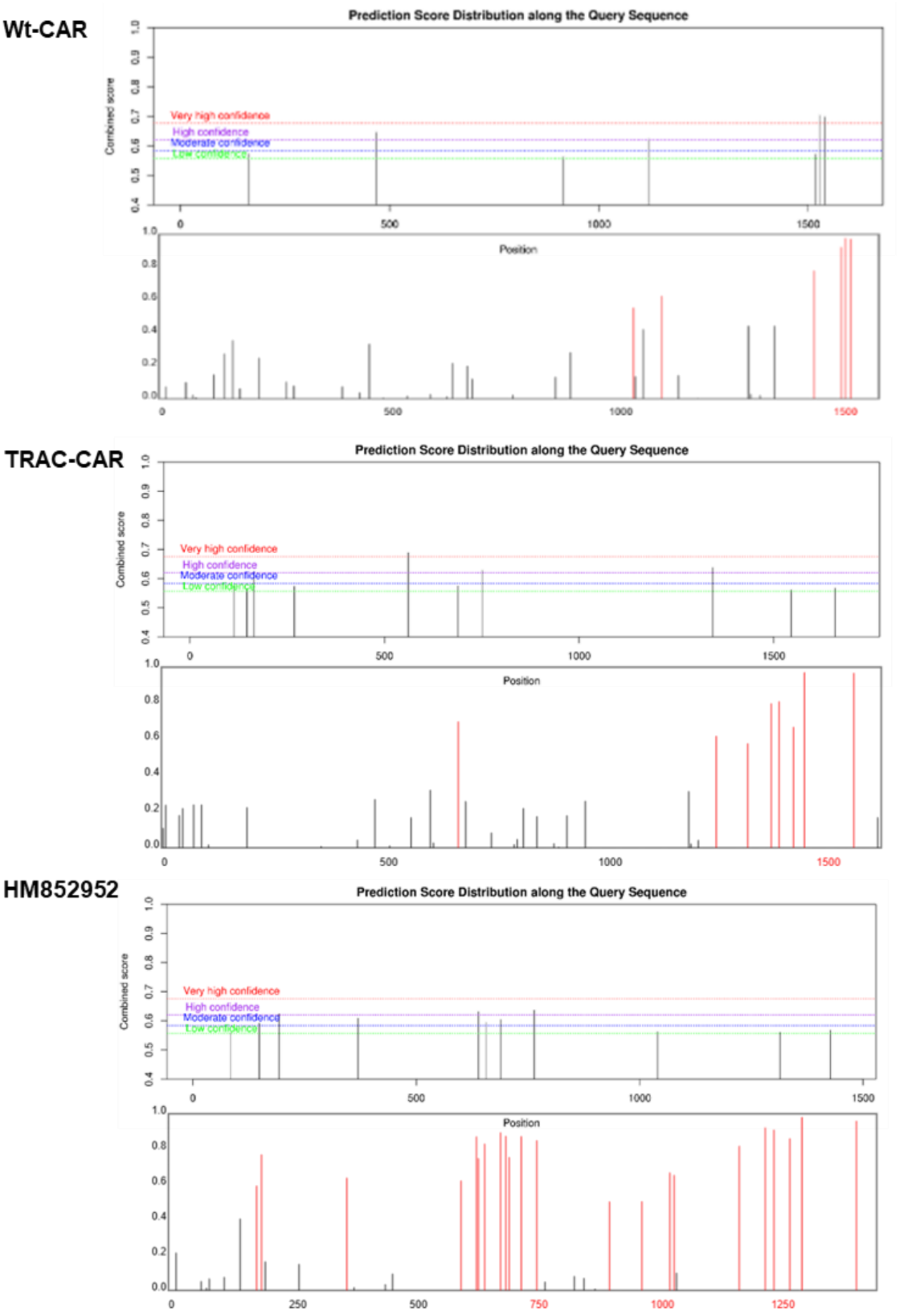

B

Wt-CAR			TRAC-CAR			HM852952		
Site	SRAMP	deepSR	Site	SRAMP	deepSR	Site	SRAMP	deepSR
			92	High	N/A	193	High	0.768
468	High	N/A	489	Very high	N/A	639	High	0.871
1118	High	0.625	1271	High	0.619	764	High	0.850
1527	Very high	0.973	1473	Low	0.971	1314	Low	0.981
1539	Very high	0.967	1586	Low	0.967	1427	Low	0.959

C

```
Wt-CAR          GGCGAGGGATCTACCAAGGGCGAAGTGAAACTGCAAGAGTCTGGCCCTGGACTGGTGGCC   477
TRAC-CAR        GGCAGCGGAGGTGGCGGCTCTGAGGTGAAACTGCAGGAGAGTGGCCCTGGCCTGGTGGCT   396
HM852952        GGCGAGGGATCCACCAAGGGCGAGGTGAAACTGCAGGAGTCAGGACCTGGCCTGGTGGCG   480
                ***   ***     *      ** ***********  ***   ** ***** ********

Wt-CAR          GTGTCCTGGATCAGACAGCCTCCTCGGAAAGGCCTGGAATGGCTGGGAGTGATCTGGGGC   597
TRAC-CAR        GTCTCCTGGATCCGGCAGCCTCCAAGAAAAGGACTGGAATGGCTGGGCGTCATCTGGGGA   516
HM852952        GTAAGCTGGATTCGCCAGCCTCCACGAAAGGGTCTGGAGTGGCTGGGAGTAATATGGGGT   600
                **   ******  * ********  * ** ** ***** ******** ** ** *****


Wt-CAR          AGCGAGACAACCTACTACAACAGCGCCCTGAAGTCCCGGCTGACCATCATCAAGGACAAC   657
TRAC-CAR        AGTGAGACCACCTACTATAATTCAGCCCTCAAGTCCCGGCTCACCATCATTAAGGACAAC   576
HM852952        AGTGAAACCACATACTATAATTCAGCTCTCAAATCCAGACTGACCATCATCAAGGACAAC   660
                ** ** ** ** ***** **    ** ** ** *** * ** ******** *********

Wt-CAR          GGATCCTGAAATGGGCGGCAAGCCCAGACGGAAGAATCCTCAAGAGGGCCTGTATAATGA   1445
TRAC-CAR        GGACCCTGAGATGGGGGGAAAGCCGAGAAGGAAGAACCCTCAGGAAGGCCTGTACAATGA   1472
HM852952        GGACCCTGAGATGGGGGGAAAGCCGAGAAGGAAGAACCCTCAGGAAGGCCTGTACAATGA   1313
                *** ***** ***** ** ***** *** ******* ***** ** ******** *****


Wt-CAR          GCTGCAGAAAGACAAGATGGCCGAGGCCTACAGCGAGATCGGAATGAAGGGCGAGCGCAG   1505
TRAC-CAR        ACTGCAGAAAGATAAGATGGCGGAGGCCTACAGTGAGATTGGGATGAAAGGCGAGCGCCG   1532
HM852952        ACTGCAGAAAGATAAGATGGCGGAGGCCTACAGTGAGATTGGGATGAAAGGCGAGCGCCG   1373
                *********** ******** *********** ***** ** ***** ********* *


Wt-CAR          AAGAGGCAAGGGACACGATGGACTGTACCAGGGACTGAGCACCGCCACCAAGGATACCTA   1565
TRAC-CAR        GAGGGGCAAGGGGCACGATGGCCTTTACCAGGGTCTCAGTACAGCCACCAAGGACACCTA   1592
HM852952        GAGGGGCAAGGGGCACGATGGCCTTTACCAGGGTCTCAGTACAGCCACCAAGGACACCTA   1433
                 ** ******** ******** ** ******** ** ** ** *********** *****
```

Figure S3. *In-silico* prediction of m6A sites in CD19-CAR transcripts. (A) Distribution of m6A sites using SRAMP (first panel) and deepSRAMP (second panel); (B) Confidence scores (high to very high) by SRAMP and prediction probability of m6A sites (at least 0.95) by deepSRAMP of m6A sites; (C) Multiple alignment with predicted m6A sites are shown. Predicted m6A sites of high confidence by SRAMP or of probability above 0.95 by SRAMP only, deepSRAMP only, or by both are shown in yellow, blue or green highlighted letters, respectively. Highlighted color scheme are the same for both (B) and (C), deepSR: deepSRAMP; High: high confidence; Low: low confidence; Very high: Very high confidence; N/A: not detected.

Transcript position (sequence_NIBRT)	OHMX20230134_001		OHMX20230134_002		OHMX20230134_003	
	Prob_mod	Mod_ratio	Prob_mod	Mod_ratio	Prob_mod	Mod_ratio
15	0.287458748	0.145518044	0.353733301	0.182608696	0.462240309	0.22403734
59	0.174893722	0.05561614	0.166414857	0.04	0.222930357	0.069967707
75	0.13204442	0.021253985	0.121772736	0.02	0.134295896	0.024210526
81	0.063237056	0.005138746	0.062082157	0	0.070040844	0.006302521
120	0.108063295	0.036997886	0.149650142	0.045627376	0.225017145	0.073954984
145	0.084419556	0.017223911	0.349716276	0.077205882	0.383615136	0.102956167
163	0.297369331	0.079617834	0.50662601	0.180451128	0.428009838	0.127481714
179	0.01806118	0.004197272	0.016885374	0.011320755	0.025065986	0.011702128
221	0.492447734	0.266591676	0.810538173	0.521235521	0.639571726	0.32996633
282	0.143364102	0.059466019	0.185338959	0.083665339	0.150879964	0.063647491
299	0.142446682	0.027892562	0.13723579	0.036423841	0.149973512	0.027689031
407	0.099559978	0.029850746	0.076449513	0.021671827	0.083450384	0.025085519
446	0.165197149	0.064881565	0.535327613	0.200573066	0.520585179	0.183222958
467	0.828873217	0.754385965	0.969964623	0.973770492	0.904690981	0.833718245
498	0.183736131	0.064724919	0.254548669	0.100890208	0.290431917	0.093103448
551	0.284629077	0.125290023	0.449812829	0.194528875	0.350822121	0.16540404
603	0.026937477	0.005291005	0.09939386	0.026737968	0.105215974	0.038812785
639	0.111609317	0.006204757	0.116346866	0.010050251	0.118361555	0.011350738
652	0.129954651	0.015957447	0.389239252	0.097014925	0.360790372	0.08813161
685	0.516974926	0.384090909	0.446857512	0.310880829	0.457095712	0.313095238
696	0.052625768	0.007159905	0.062795132	0.010126582	0.061803345	0.007281553
786	0.130292982	0.049197861	0.243718877	0.099537037	0.252221286	0.098676294
881	0.033328481	0.006185567	0.250287265	0.069473684	0.128138363	0.033603708
914	0.772509098	0.552109181	0.98699683	0.862842893	0.915313601	0.700268817
1054	0.344538927	0.161764706	0.529284239	0.231707317	0.34957099	0.143705463
1059	0.057583809	0.001199041	0.069298856	0.00390625	0.062627763	0.004021448
1076	0.265540928	0.118995633	0.40015018	0.1625	0.373611271	0.150510204
1117	0.142919809	0.053030303	0.722569108	0.404513889	0.50977385	0.226460072
1154	0.115786523	0.047034765	0.282473505	0.090332805	0.355531007	0.110593714
1197	0.062593423	0.00547046	0.073127709	0.009302326	0.085105918	0.009779951
1310	0.506165206	0.242690058	0.910229385	0.481404959	0.83065027	0.3808
1315	\	\	0.084888838	0.032258065	0.035301134	0.007272727
1336	\	\	0.176152363	0.051502146	0.234572694	0.064516129
1369	\	\	0.522647321	0.244285714	0.509044409	0.238153098
1456	\	\	0.209146962	0.067975831	0.209825397	0.060102302
1517	\	\	0.83194387	0.290066225	0.61855197	0.146282974
1526	\	\	0.945919573	0.977578475	0.888751209	0.777173913
1538	\	\	0.994802237	0.98343949	0.980043232	0.930288462

Figure S4. m6A sites on CD19-CAR by nanopore RNA direct sequencing (n = 1). Probability of modification (Prob-mod) and Modification Ratio (Mod-ratio) of m6A methylation at the predicted DRACH sites on CD19-CAR by nanopore sequencing in UT-IVT-CAR (OHMX20230134_001), LV-CAR (OHMX20230134_002) and IVT-CAR (OHMX20230134_003). Note that the software - m6Anet - tool uses zero-based indexing to report these positions. This means that the position starts with 0. For comparison of these coordinates to the one-based indexing software (e.g. SRAMP), 1 should be added to each position.

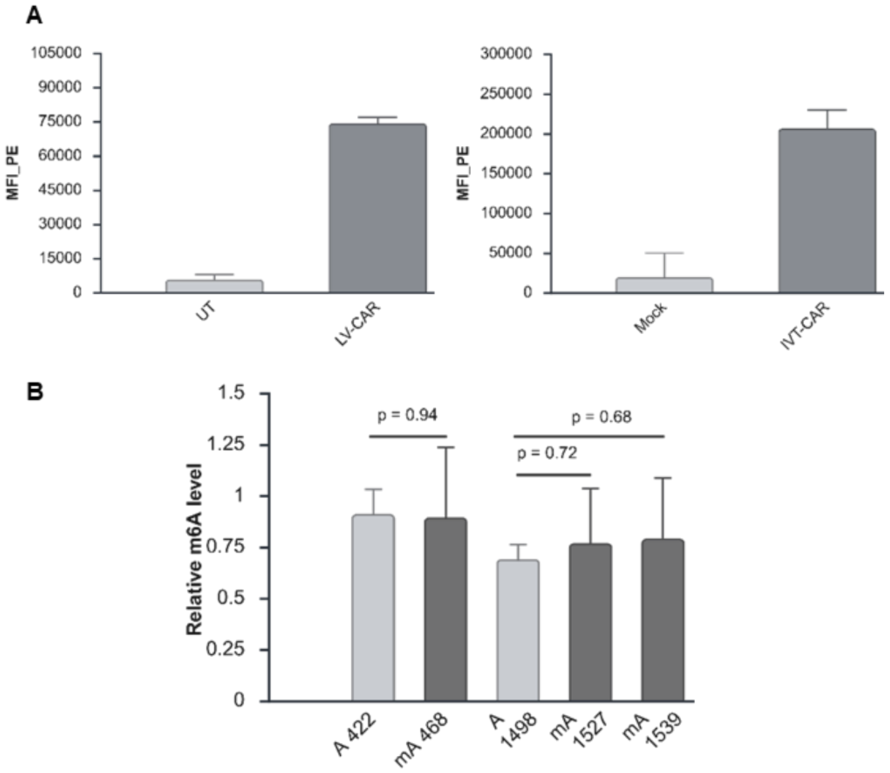

Figure S5. Expression of LV-CAR and IVT-CAR and relative m6A levels in the UT-IVT-CAR control. (A) Relative Median Fluorescence Intensity (MFI) of LV-CAR (left panel) and IVT-CAR (right panel); n = 3 biological replicates; n = 3 technical replicates; (+/-SD); (B) Relative m6A levels at the target sites nt468, nt1527 and nt1539 compared to the control sites A422 and A1498 in UT-IVT-CAR transcript with spiked-in IVT mRNA of wt-CAR using m6A-qPCR (n = 2 biological replicates; n = 3 technical replicates); (+/-SD); ns (p>0.05) (two-sided Student's *t*-test). Created at https://BioRender.com.

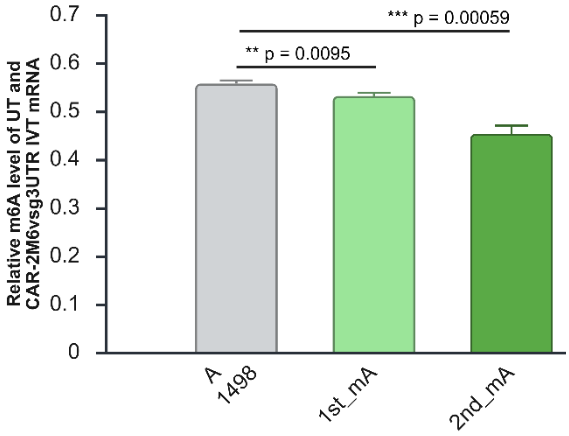

Figure S6. Relative m6A levels at the first, second consensus m6A sites and the control A site A 1498 in the Untreated activated T cells with added IVT mRNA CAR-2M6vsg3UTR (n = 2 biological replicates; n = 3 technical replicates); (+/-SD); ** indicates $p<0.01$, and *** indicates $p<0.001$ (Two-sided Student's *t*-test); first (1st); second (2nd). Created at https://BioRender.com.

A. Reagents and kits

Reagents and kits	Identifier	Source
TexMACS™	130-097-196	Miltenyi Biotech
Human IL-7, premium grade	130-095-361	Miltenyi Biotech
Human IL-15, premium grade	130-095-764	Miltenyi Biotech
CD19 CAR Detection reagent, human, biotin	130-129-550	Miltenyi Biotech
Biotin Antibody, PE, REAfinity™	130-111-068	Miltenyi Biotech
T cell Tract Act_Human	130-128-758	Miltenyi Biotech
7-AAD Staining Solution	130-111-568	Miltenyi Biotech
CD3 Antibody, anti-human, FITC	130-113-138	Miltenyi Biotech
CD25 Antibody, anti-human, PE	130-113-286	Miltenyi Biotech
Invitrogen™ SYTOX™ Deep Red Nucleic Acid	S11380	Invitrogen
FITC-Labelled Monoclonal Anti-FMC63 Antibody	FM3-FY45	AGROBiosystems
DNA Clean & Concentrator™-25	D4033	Zymo Research
Direct-zol™ RNA MiniPrep kit	R2050	Zymo Research
High-Yield T7 ARCA mRNA Synthesis Kit	RNT-102-L	Jena
Invitrogen™ Qubit™ RNA High Sensitivity kit	Q32852	Thermo Fisher Scientific
P3 Primary Cell 4D- Nucleofector X kit S	V4XP-3032	Lonza
Gibco™ PBS, pH 7.2	20012068	Gibco
Invitrogen™ TRIzol™ Reagent	15596026	Invitrogen
Bst 2.0® DNA Polymerase	M0537S	New England Biolabs
Monarch RNA clean-up kit	T2050S	New England Biolabs
DNAse I	M0303S	New England Biolabs
Invitrogen™ SuperScript™ IV Reverse Transcriptase	18090010	Invitrogen
Applied Biosystems™ PowerUp™ SYBR™ Green Master Mix	A25742	Applied Biosystems
Invitrogen superRNase RNA inhibitor	AM2694	Invitrogen
SYBR™ Fast SYBR Green Master Mix	10459604	Fisher Scientific
Thermo Scientific™ RIPA Lysis and Extraction Buffer	89901	Thermo Fisher Scientific
Thermo Scientific HALT ™ Protease Inhibitor Cocktail	78429	Thermo Fisher Scientific
Invitrogen™ NuPAGE™ MOPS SDS Running Buffer (20X)	NP0001	Invitrogen
Thermo Scientific™ Pierce™ 1-Step Transfer Buffer	84731	Thermo Fisher
Nitrocellulose membrane (Amersham ™ Protran ™ 0.45um)	*10600002*	Amersham
STM2457	HY-134836	MedChemExpress
High-Capacity cDNA synthesis	4368814	Applied Biosystems™
Fast SYBR™ Green Master Mix	4385616	Applied Biosystems™
**Primary and Secondary for Western blots**		
CD247 polyclonal antibody	12837-2-AP	Proteintech
GAPDH Mouse Monoclonal antibody	60004-1-IG	Proteintech
Rabbit anti-METTL3/MT-A70 Antibody	A301-567A-T	Abcam
IRDye® 800CW Goat anti-Mouse IgG	P/N 925-32210	Licorbio
IRDye® 680LT Goat anti-Mouse IgG	P/N 925-68020	Licorbio
IRDye® 800CW Goat anti-Rabbit IgG	P/N 926-32211	Licorbio
IRDye® 680RD Goat anti-Rabbit IgG	P/N: 926-68071	Licorbio

B. **Primers for qPCR (from Integrated DNA Technologies at https://eu.idtdna.com/**

**Primer names**	**Primer sequence (5′ to 3′ direction)**
467_CadjR	CGCTCAGAGACTGAGATGGGGCCACCAG
467-CnonadjR	GCCAGACTCTTGCAGTTTCACTTCG
467_F	GCTGGAAATCACCGGCTCTACAAG
A422AdjR	GTTTCACTTCGCCCTTGGTAGATCCC
A391AdjR	ATCCAGGTTTGCCGCTGCCGCTTG
331F	CAAGGCAACACCCTGCCTTACACC
1527-CadjR	GGTGGCGGTGCTCAGTCCCTGGTACAG
1527_CminusR	CCTTCATTCCGATCTCGCTGTAGGC
1527_F	GATCCTGAAATGGGCGGCAAGC
1539_CadjR	CATAGGTATCCTTGGTGGCGGTGCTCAG
A1498AdjR	TCGTGTCCCTTGCCTCTTCTGCGC
GS32_2ndm6A_PlusR	GTAGCAAGAATTTTTAAGAAAAAGAGGGGGAAAAGGTGGCACAAG
GS32_1stm6A_PlusR	GCAAGAATTTTTAAGAAAAAGAGGGGGAAAAGGTGGCACAAGTCCGCTTT AG
GS32_minusR	CTTTAGTCCTTTATCTTGGAGGCAGGGCC
GS32_F	GCAGAAGAGGCAAGGGACACGATG
GS33_2ndm6A_PlusR	GTGTTAAAATATATCAGGAGTTTTCAG
GS33_1stm6A_PlusR	GTGTTAAAATATATCAGGAGTTTTCAGTCCGCTTTAG
GS33_MinusR	GAGGGGGAAAATTTATCTTGGAGGCAGGGCCTGCA
GS33_F	GAGCACCGCCACCAAGGATACCTATG
19CAR_489F	TCTGAGCGTGACCTGTACAG
19CAR_624R	GGCGCTGTTGTAGTAGGTTG
GAPDH-HuF	CAAATTCCATGGCACCGTCA
GAPDH-HuR	ATCGCCCCACTTGATTTTGG

Table S1. Reagents and Primers. (**A**) Reagents and kits; (**B**) Primer sequences for qPCR.
