# Supplementary figures and images for: m6A-Modified Nucleotide Bases Improve Translation of In Vitro-Transcribed Chimeric Antigen Receptor (CAR) mRNA in T Cells
Source: Int J Mol Sci. 2026 Jan 13;27(2):796. doi: 10.3390/ijms27020796 (PMC12841529; doi:10.3390/ijms27020796)

## FLOW PLOTS\_MFI-PE- 48 hours\_Figure 2C

| Names on plots | Names on paper |
|----------------|----------------|
| Wt             | wt-CAR         |
| Mut            | mut-CAR        |

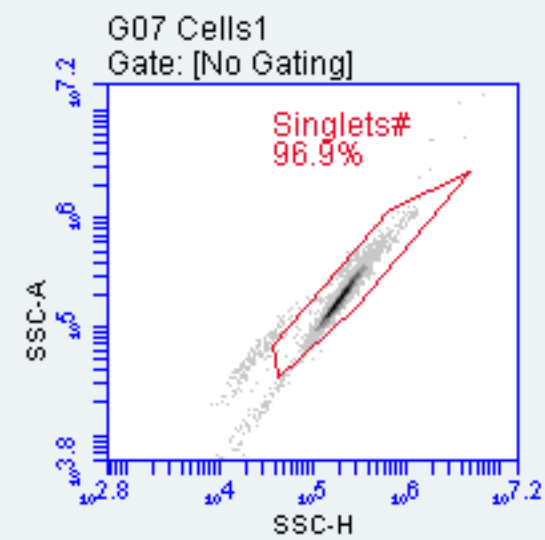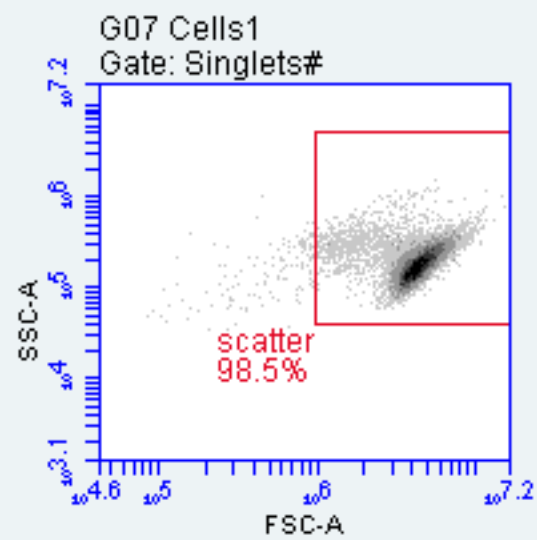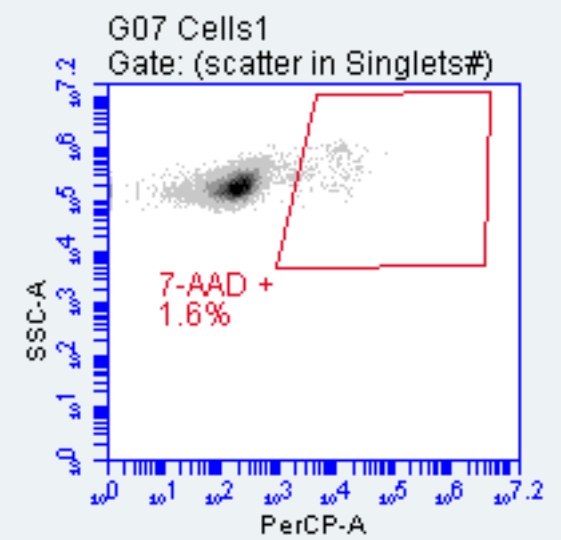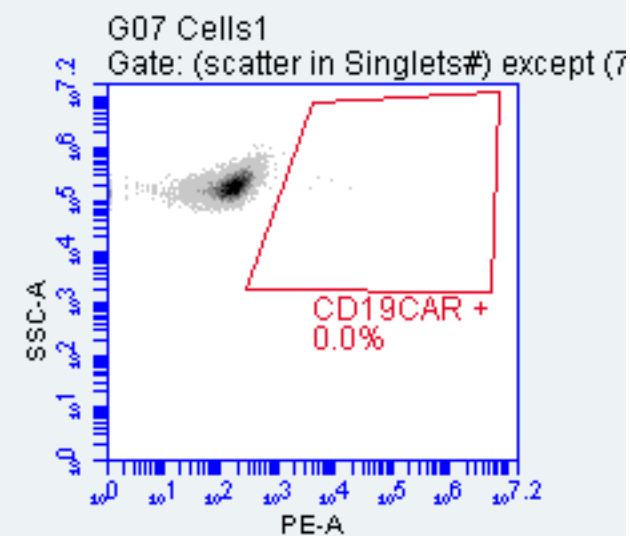

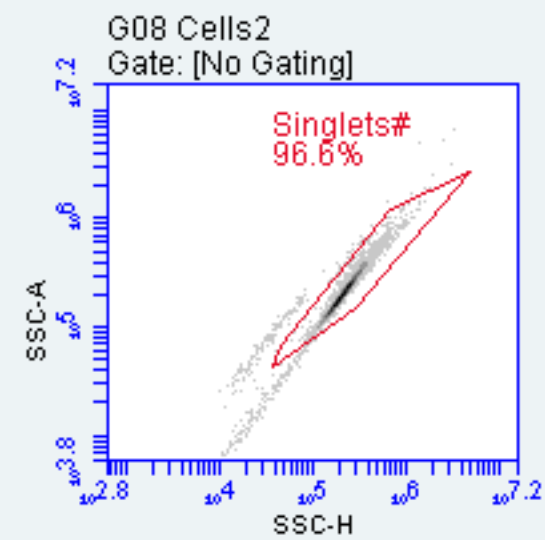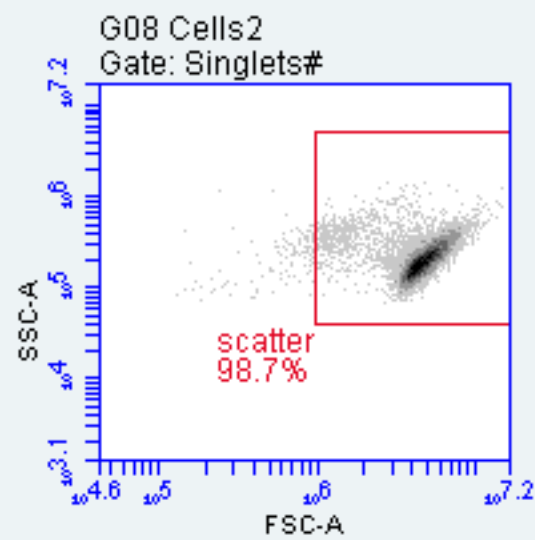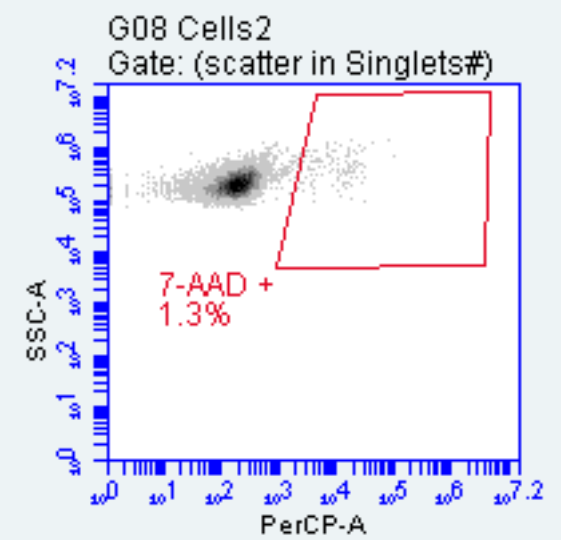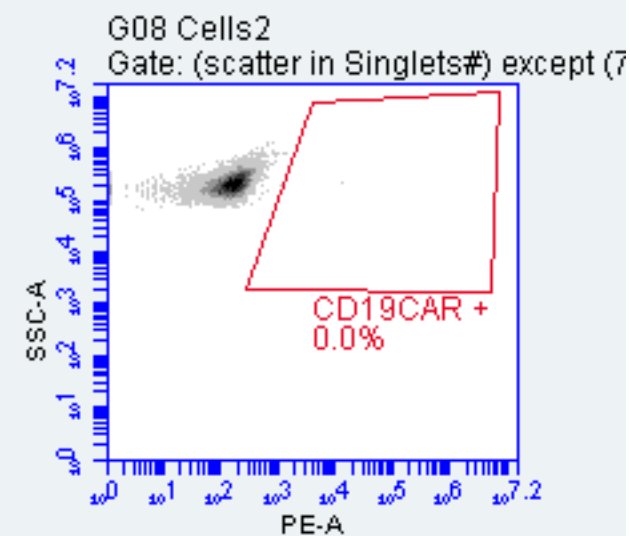

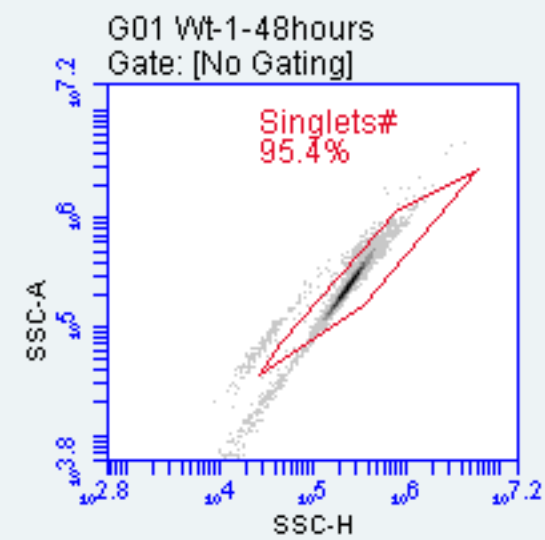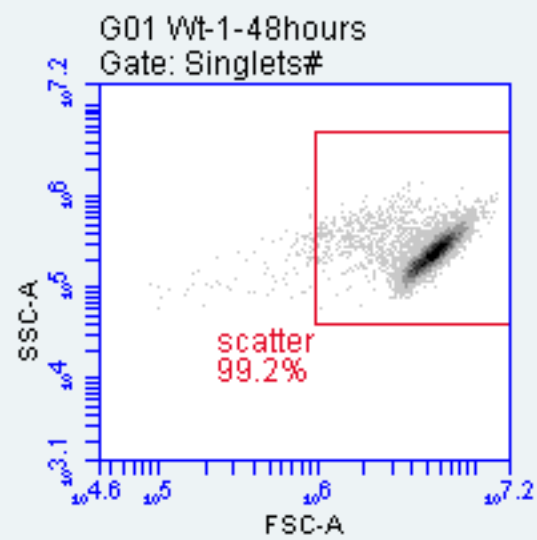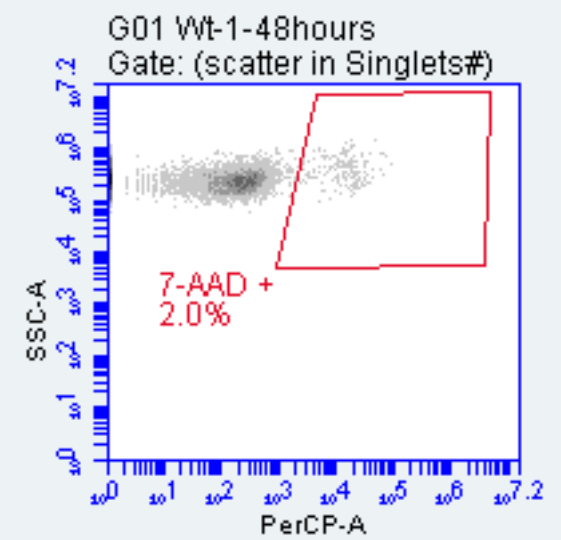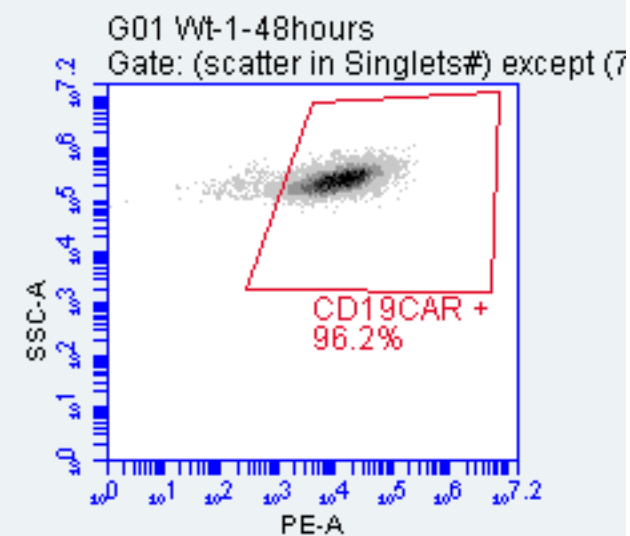

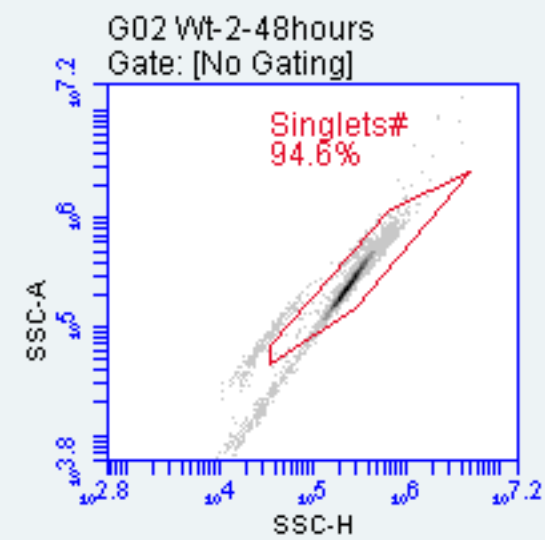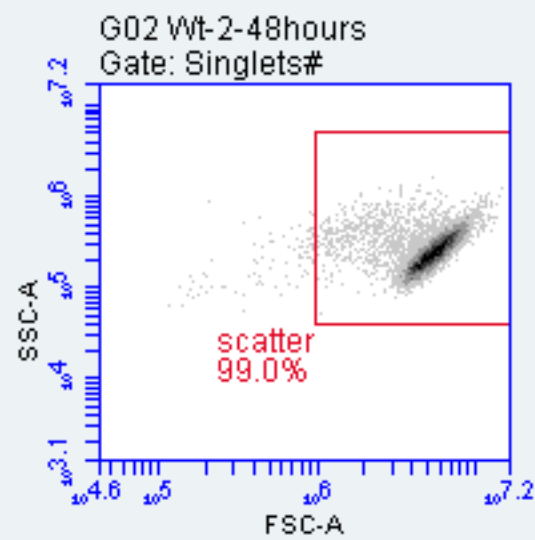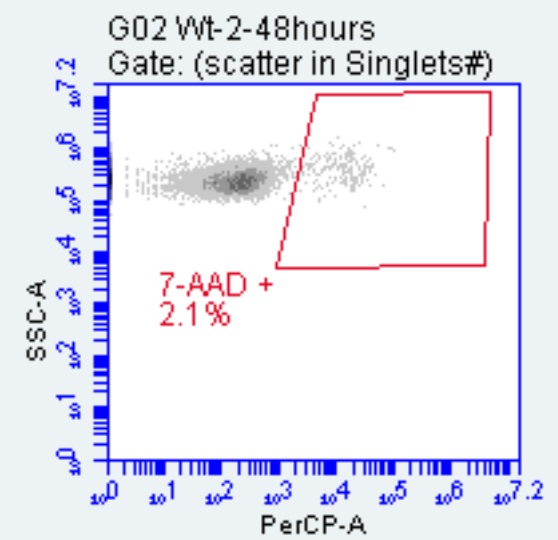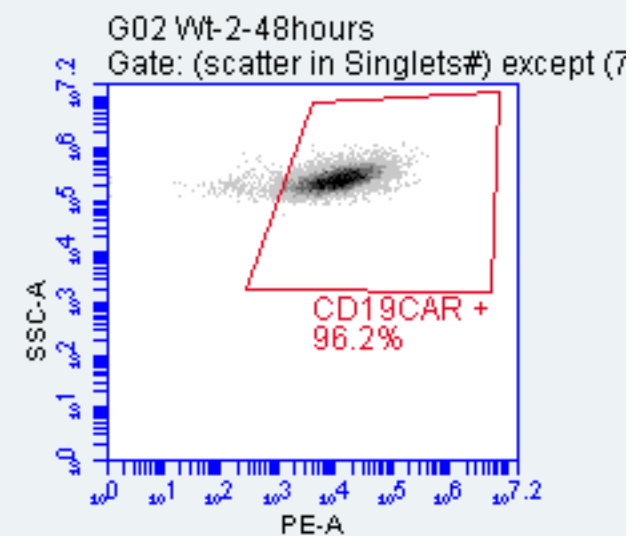

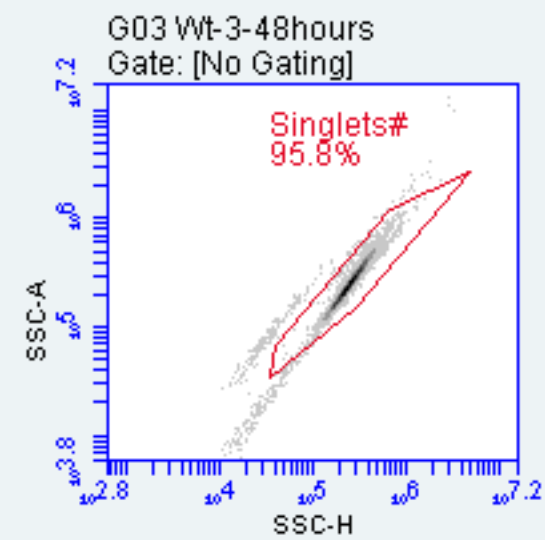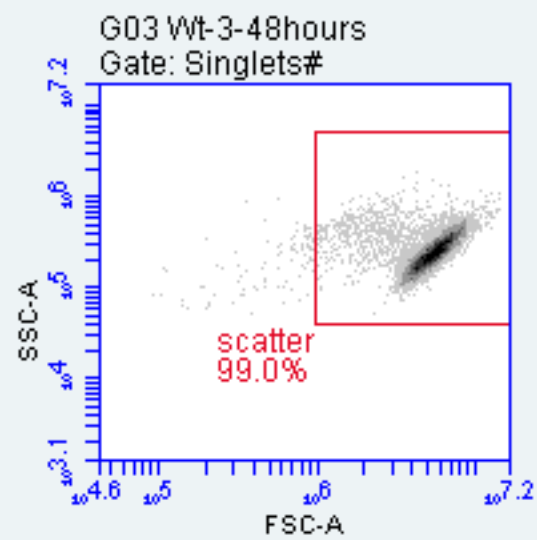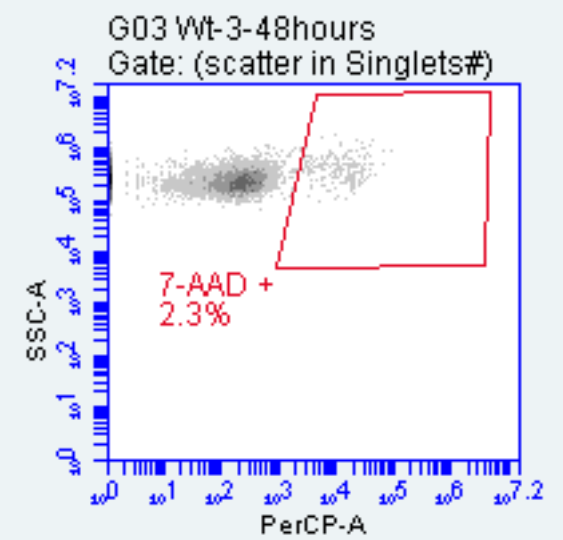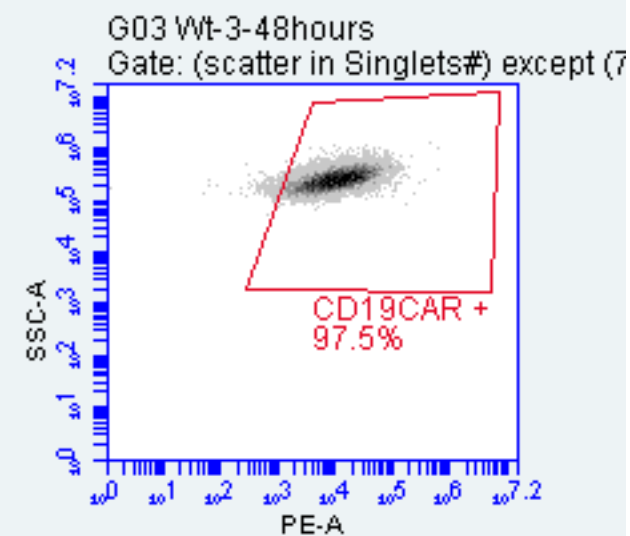

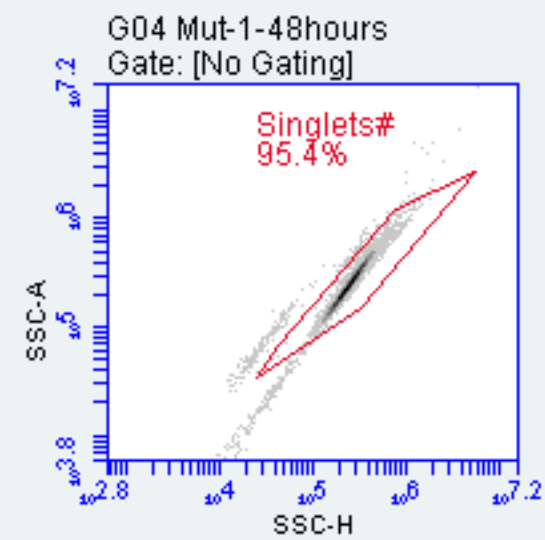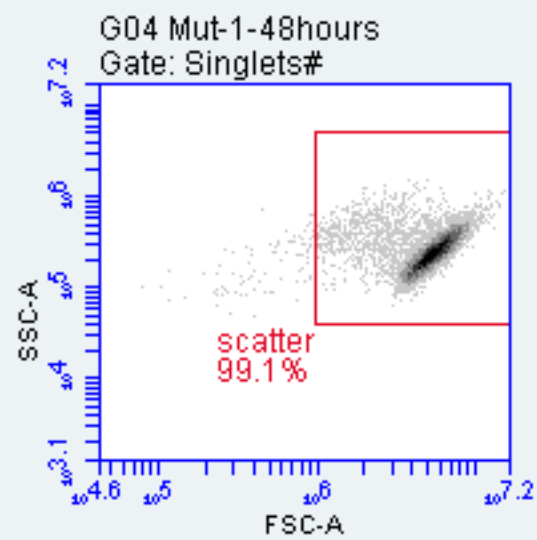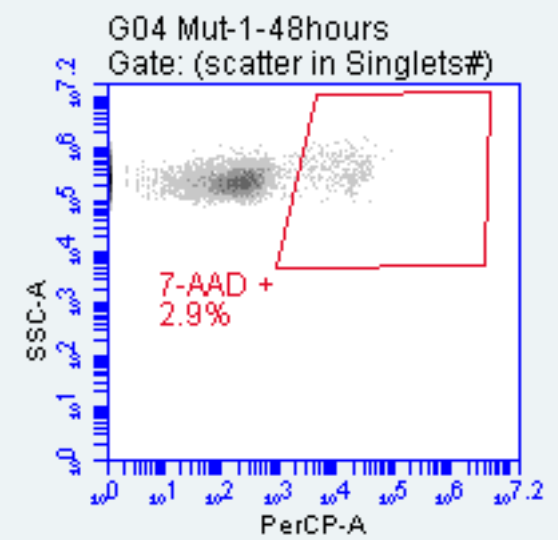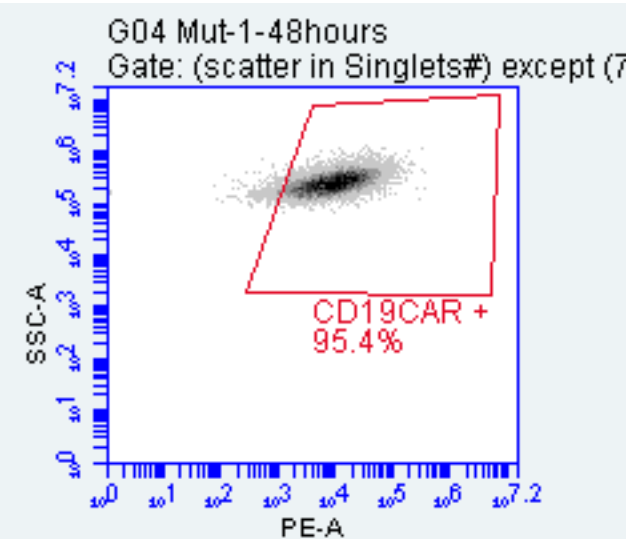

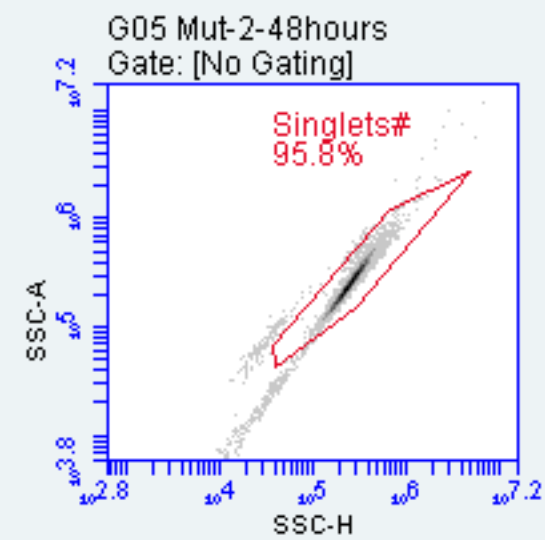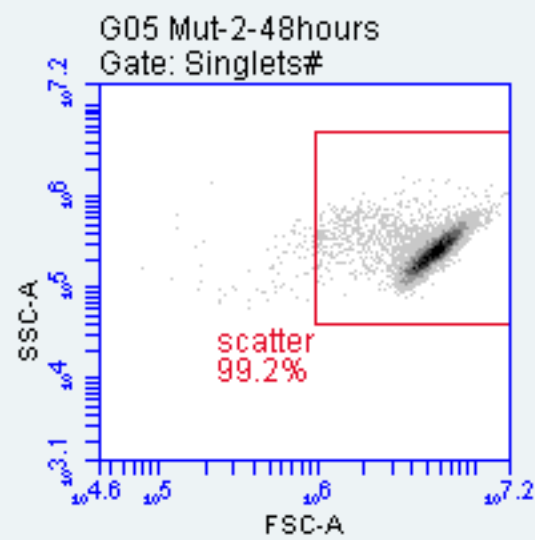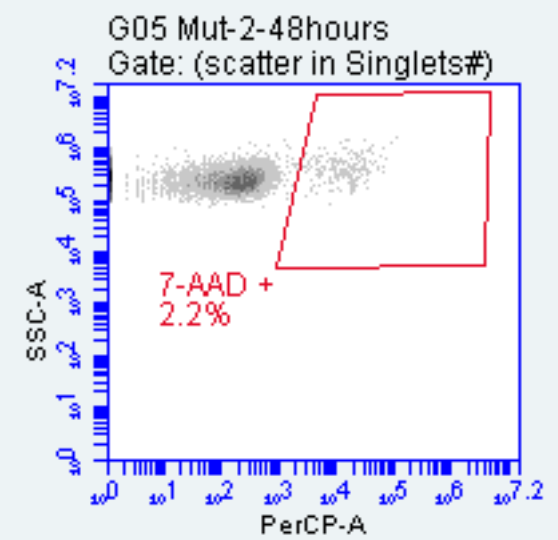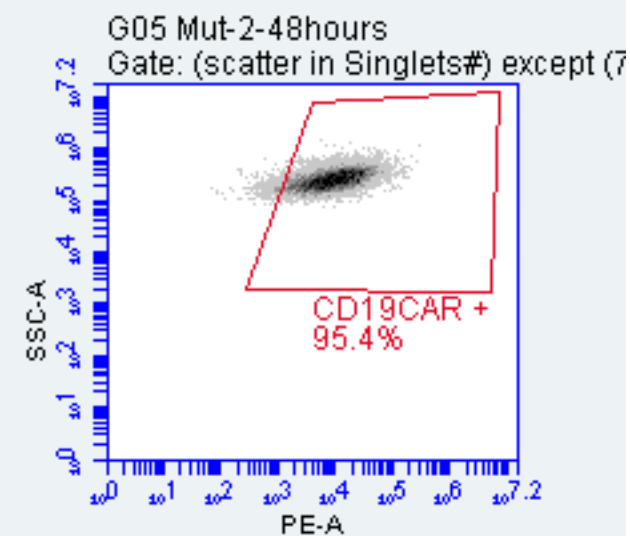

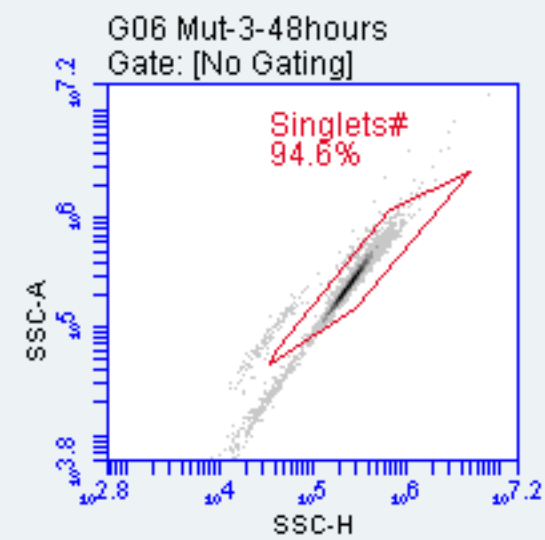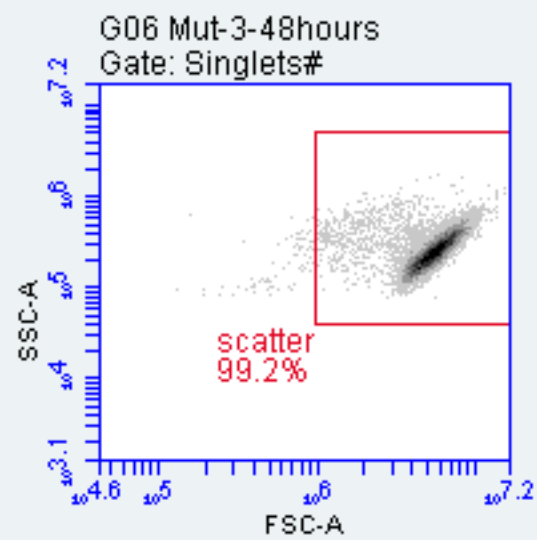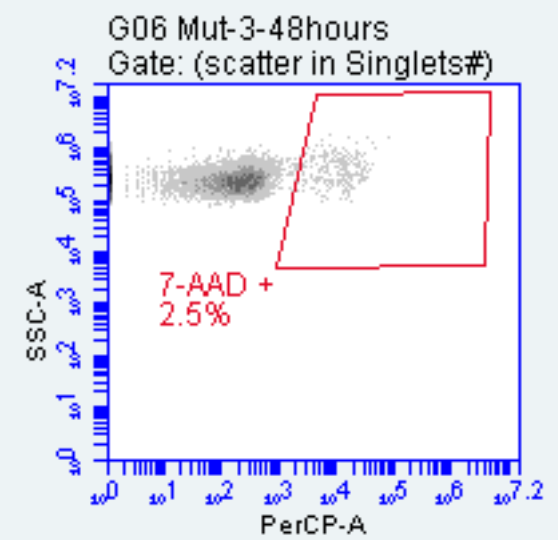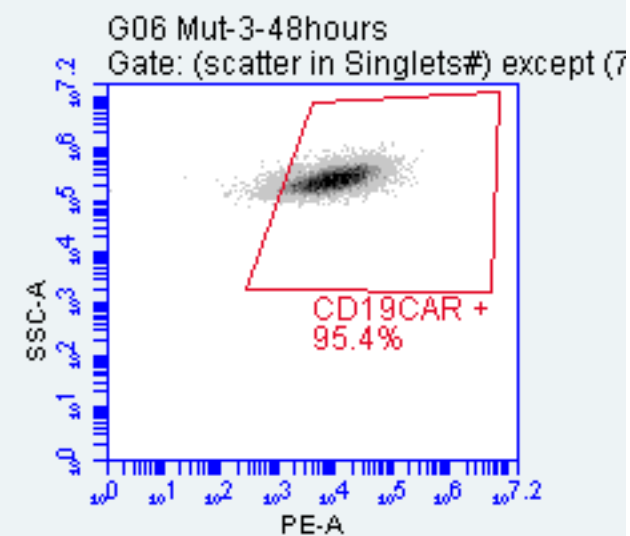

Supplement: Supplementary file 1 [file ijms-27-00796-s001.zip › Suppl_flow plot example.pdf]
